# Stimuli‐Responsive Biomaterials: Scaffolds for Stem Cell Control

**DOI:** 10.1002/adhm.202001125

**Published:** 2020-09-30

**Authors:** Amy Gelmi, Carolyn E. Schutt

**Affiliations:** ^1^ School of Science College of Science, Engineering and Health RMIT University Melbourne VIC 3001 Australia; ^2^ Department of Biomedical Engineering Knight Cancer Institute Cancer Early Detection Advanced Research Center (CEDAR) Oregon Health and Science University Portland OR 97201 USA

**Keywords:** biomaterials, electrical stimulation, magnetic fields, photostimulation, stem cells, stimuli‐responsive materials, ultrasound

## Abstract

Stem cell fate is closely intertwined with microenvironmental and endogenous cues within the body. Recapitulating this dynamic environment ex vivo can be achieved through engineered biomaterials which can respond to exogenous stimulation (including light, electrical stimulation, ultrasound, and magnetic fields) to deliver temporal and spatial cues to stem cells. These stimuli‐responsive biomaterials can be integrated into scaffolds to investigate stem cell response in vitro and in vivo, and offer many pathways of cellular manipulation: biochemical cues, scaffold property changes, drug release, mechanical stress, and electrical signaling. The aim of this review is to assess and discuss the current state of exogenous stimuli‐responsive biomaterials, and their application in multipotent stem cell control. Future perspectives in utilizing these biomaterials for personalized tissue engineering and directing organoid models are also discussed.

## Graphical abstract

Stimuli‐responsive biomaterial systems are a powerful tool to deliver temporal and spatial cues to stem cells, and ultimately control stem cell fate. This review presents recent developments in exogenous stimuli‐responsive biomaterial scaffolds—engineered to respond to light, electric, ultrasound, and magnetic triggers—and their application to stem cell manipulation, additionally highlighting future applications to personalized tissue engineering and organotypic models.

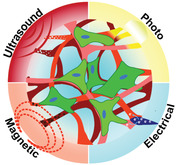

## 1 Introduction

Biomaterial platforms to regulate and control stem cell fate is an area of tissue engineering that has grown exponentially over the last 15 years,^[^ ^1^, ^2^ ^]^ beginning with research demonstrating that a material property as simple as mechanical stiffness directs the lineage of mesenchymal stem cells.^[^ ^3^ ^]^ As the field has progressed, it is increasingly more apparent that these biomaterial systems can be far more than just a passive support for cells—through careful and thoughtful design and fabrication we can introduce temporal and spatial control of biomaterial properties, enabling targeted stimulation of stem cells.^[^ ^4^, ^5^, ^6^ ^]^ Living tissue is a dynamic and changing environment, and recapitulating this dynamic environment for stem cells in a fabricated biomaterial scaffold can greatly enhance our ability to control stem cell fate.^[^ ^5^ ^]^

There are many endogenous cues that play an important role in the extracellular microenvironment for stem cells: changing stiffness as tissue matures,^[^ ^7^ ^]^ electrical fields generated by piezoelectric forces or cell–cell signaling in the tissue,^[^ ^8^, ^9^ ^]^ strains and shear flow, and growth factors and signaling molecules.^[^ ^10^, ^11^ ^]^ The temporal nature of these endogenous signals is an important factor in their efficacy, hence creating biomaterial scaffolds which can mimic these signals via a controlled, exogenous stimulus enables a more sophisticated and adaptable approach to manipulating stem cell behavior.

**Figure 1:**
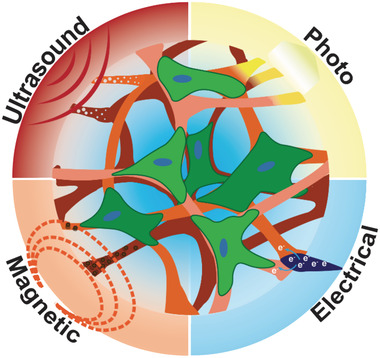
Stimuli‐responsive scaffolds transduce exogenously applied signals to stimulate stem cells. These externally applied stimuli can include light, electrical stimulation, ultrasound, and magnetic fields.

Stimuli‐responsive biomaterials can encompass a broad range of external signals, however the most promising and versatile are those capable of transducing an external energy source into a meaningful signal for cells with minimal design complexity. These external signals include photostimulation, electrical stimulation, ultrasound activation, and magnetic field stimulation (**Figure** **1**). Each of these stimuli can be applied directly to a biomaterial scaffold to induce a change or response, which in turn will convert the stimuli into a meaningful trigger for the cells. The advantage of this approach is that the stimulus can be controlled both temporally and spatially, enabling precise control over the cellular response as required. Furthermore, the extent of the biomaterial response (e.g., quantity of molecular release, strain) can be tuned by the magnitude of the applied external signal, enabling far greater control than a simple binary switch.

While some stimuli‐responsive biomaterials, particularly the photoresponsive biomaterials, have had a large amount of focus across the field of tissue engineering, there are others which have been less widely explored for the control of stem cell fate. For example, electrical stimulation has been used with great effect in the areas of neural and nerve stimulation but less so for multipotent stem cell differentiation.^[^ ^12^ ^]^ There has been promising research demonstrating that electrical stimulation can have a marked effect on stem cells,^[^ ^13^ ^]^ and so using conductive biomaterial scaffolds to deliver electrical cues to cells is being increasingly explored. Ultrasound stimulation has been previously utilized for controlled in vivo drug delivery due to its significant tissue penetration and remote actuation capabilities,^[^ ^14^ ^]^ and shows potential as a versatile stimulus for remote material manipulation. Magnetically responsive biomaterial scaffolds are another interesting system, wherein this type of responsive platform has been used to great effect in nanomedicine,^[^ ^15^ ^]^ and is easily applied whether in vitro or in vivo.

Our interest here lies in assessing stimuli‐responsive biomaterials for the control of stem cell fate, and where possible, multi‐ or pluri‐potent stem cells. Progress in the field of tissue engineering is moving toward personalized medicine; the ability to tailor engineered tissue or in vitro disease model platforms for individual patients using their own stem cells is an exciting prospect, which can reduce therapeutic risks and greatly increase efficacy. By identifying the most promising biomaterial and external stimulation combinations for different tissue types, precisely controllable tissue‐engineered platforms can be designed and leveraged to drive advances in biomedicine.

## 2 Photostimulation

**Figure 2:**
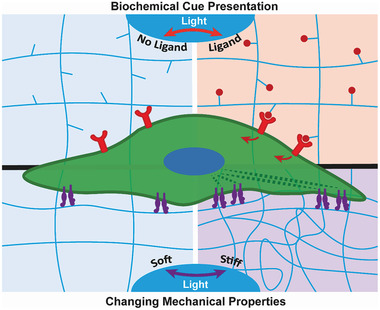
Schematic of cell stimulation using photoresponsive materials. Top: Light exposure can be used to spatiotemporally modulate the presentation of biochemical cues, including growth factors and adhesion peptides. Bottom: Photostimulation can also modulate the mechanical properties (e.g., stiffness) of the scaffold matrix to induce cellular response via integrin‐mediated mechanotransduction. Integrins (purple) interact with the scaffold matrix and transduce matrix mechanical properties via the actin cytoskeleton.

Light is a versatile stimulus for manipulation of stem cells via responsive biomaterial scaffolds due to the wide range of available photochemical linkers,^[^ ^16^, ^17^, ^18^ ^]^ as well as the ability to localize the applied energy. Spatial patterning of scaffolds can be achieved through the use of photomasks or by focusing the light in three dimensions to create complex geometries.^[^ ^19^ ^]^ Further manipulation over time can be applied to create dynamic cell environments, a concept referred to as “4D patterning.”^[^ ^16^, ^20^, ^21^ ^]^ There exists an extensive library of photoresponsive chemical moieties which can be activated by various wavelengths in the visible, ultraviolet, and near‐infrared range, resulting in cleavage or structural change.^[^ ^17^, ^22^ ^]^ Many of these compounds have been previously utilized in the design of photoactivated drugs and drug delivery vehicles.^[^ ^23^, ^24^, ^25^, ^26^ ^]^ The incorporation of photoactive chemical compounds within biocompatible scaffold materials, such as hydrogels, can allow spatially selective or bulk manipulation of scaffold properties,^[^ ^6^ ^]^ thereby controllably changing the stem cell microenvironment.^[^ ^2^, ^27^ ^]^ Light stimulation can be used to define the presentation of biochemical cues as well as tune mechanical properties of the substrate (**Figure** **2**). Several photocontrolled scaffold systems which use these strategies for stem cell manipulation are summarized in **Table** **1**. Through the development of responsive scaffolds with well‐defined photochemical elements, light can be used to control a wide range of cell properties and behaviors including migration, adhesion, and differentiation.

**Table 1:** Photoresponsive materials for stem cell manipulation via biochemical cue presentation or changing mechanical properties

Material	Photostimulus wavelength [nm]	Material response	Cell type	Cell response	Reference
Biochemical cue presentation					
PEG hydrogels with photocaged (Nvoc) peptide substrate of ECM crosslinking enzyme	405	Uncaging of peptide substrate allows localized enzymatic biomolecule tethering (RGD, FN_9–10_, PDGF‐BB)	hMSC	Directed migration	^[^ ^30^ ^]^
PEG hydrogel crosslinked with allyl sulfide functional groups, swollen in photoinitiator (I2959)	720 (two‐photon)	Laser‐initiated thiyl radical production allows cysteine‐terminated ligand (RGD) attachment/exchange through thiol‐ene click chemistry	hMSC	Cell attachment	^[^ ^32^ ^]^
Agarose hydrogel with photocaged (coumarin) thiols	740 (two‐photon)	Uncaging and reaction with maleimide‐conjugated linkers to immobilize stem cell differentiation factors (SHH, CNTF)	Retinal precursor cells (RPC), neural precursor cells (NPC)	Directed migration (of NPC)	^[^ ^33^ ^]^
PEG hydrogel with RGDS adhesion peptide attached via photolabile tether (nitrobenzyl ether‐derived)	365	RGDS photocleavage and removal on day 10	hMSC	Increased chondrogenic differentiation (GAG, type II collagen production)	^[^ ^34^ ^]^
PEG hydrogel with BMP‐2 (nitrobenzyl tether) and BMP‐7 (coumarin methylester tether) attached via photolabile tethers	405, 365 (sequentially timed exposure)	BMP‐2 release (day 1), BMP‐7 release (day 4)	hMSC	Increased ALP activity (osteogenic response)	^[^ ^35^ ^]^
PEG hydrogel with photocaged (NPPOC) alkoxyamines	365 or 740 (single or multiphoton)	Photocontrolled attachment of functionalized vitronectin via oxime ligation and photocontrolled release (via *ortho*‐nitrobenzyl ester linker)	hMSC	Spatially defined, reversible differentiation to osteoblasts (OCN, ALP activity)	^[^ ^36^ ^]^
Photoinduced mechanical property changes					
PEG‐based hydrogel with nitrobenzyl ether‐containing photodegradable crosslinker (PEGdiPDA)	365	Hydrogel degradation and stiff to soft transition	hMSC	Deactivation of preosteogenic markers (YAP, RUNX2) unless precultured on stiff substrate beyond temporal threshold of irreversibility	^[^ ^50^ ^]^
PEG‐based hydrogel with allyl sulfide crosslinker, swollen in photoinitiator (LAP) with glutathione	365	Radical‐mediated AFCT replacing crosslinking allyl sulfides with non‐crosslinked counterparts resulting in stiff to soft transition	hMSC	Reversal of increased histone acetylation and decreased chromatin condensation, unless precultured on stiff substrate beyond temporal threshold of irreversibility	^[^ ^51^ ^]^
Methacrylate‐modified HA hydrogels swollen with photoinitiator (Irgacure 2959)	365	Gel stiffening via radical polymerization	hMSC	Earlier stiffening promotes osteogenesis; later stiffening promotes adipogenesis (cultured in bipotential medium)	^[^ ^54^ ^]^

### 2.1 Biochemical Cue Presentation

**Figure 3:**
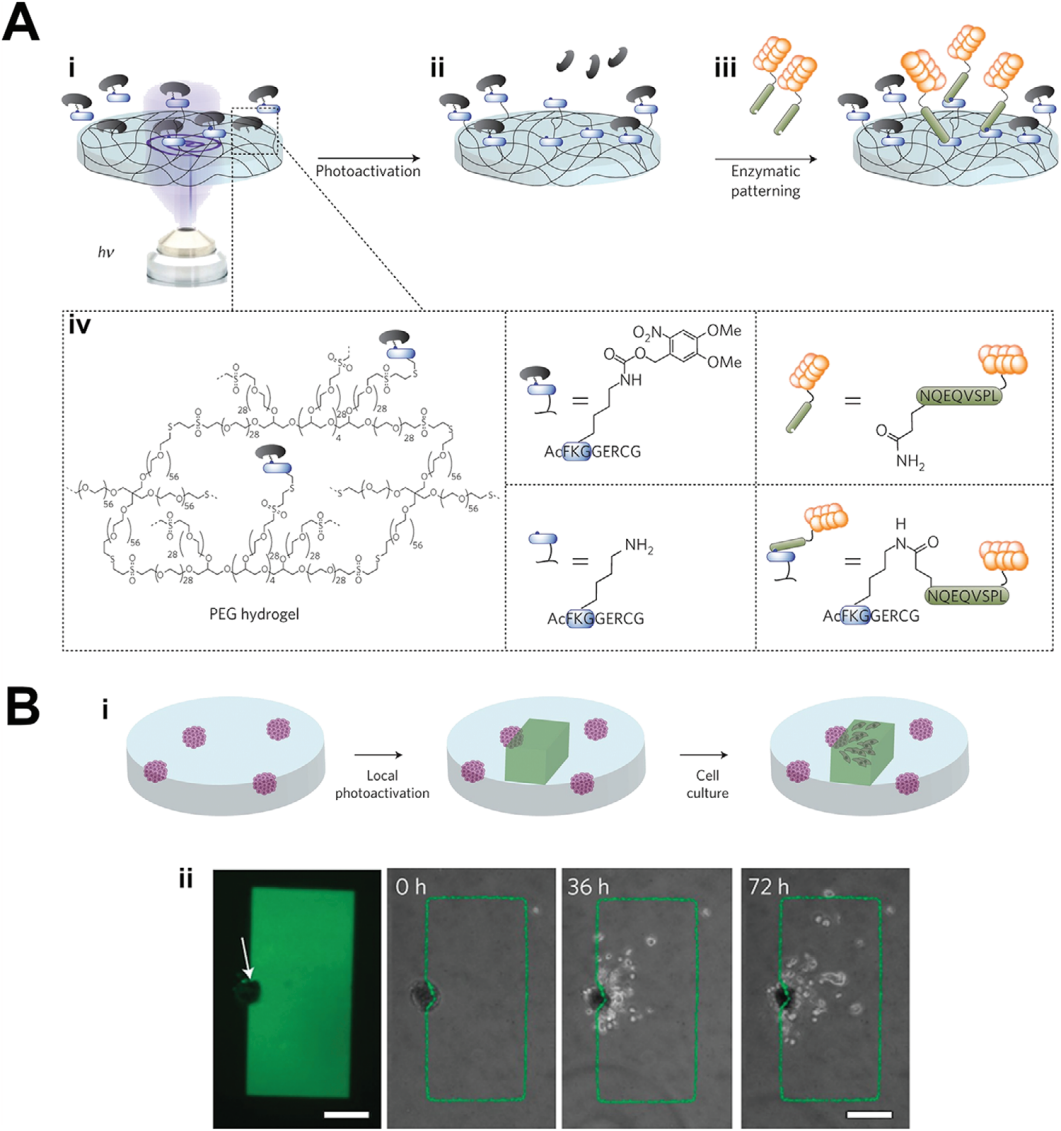
Photocontrolled biochemical cue presentation: chemical cues to influence cell migration. A) Schematic of photocontrolled, enzyme‐mediated biomolecule patterning of hydrogels. Hydrogels incorporate i,iv) a photocaged peptide substrate which can be ii) photoactivated to allow iii) enzyme‐catalyzed conjugation with the biomolecule of interest, forming a light‐defined pattern. B‐i) hMSC microtissues within a PEG hydrogel which is photopatterned with fluorescent adhesion peptide to guide hMSC invasion in the 3D gel. ii) Confocal image (left) and image series of hMSC invading into the patterned fluorescent RGD adhesion peptide region (green). Scale bar 200 µm. Adapted with permission.^[^ ^30^ ^]^ Copyright 2013, Springer Nature.

Stem cells are influenced by the local presentation of biochemical ligands, including growth factors and peptides.^[^ ^10^, ^28^, ^29^ ^]^ Photoresponsive scaffold design can be leveraged to control the spatial patterning of these cues, leading to defined signaling regions which influence cell behavior or fate. A study by Mosiewicz et al. utilized an enzymatic photopatterning mechanism to direct human mesenchymal stem cell (hMSC) migration.^[^ ^30^ ^]^ A peptide substrate of extracellular matrix (ECM) crosslinking enzyme FXIIIa was inactivated by photocaging with nitroveratryloxycarbonyl (Nvoc) and incorporated into poly(ethylene glycol) (PEG) hydrogels (**Figure** **3**A). Photoactivated uncaging of the peptide substrate enabled enzyme‐catalyzed biomolecule tethering in a localized fashion to the 3D region of interest defined by the ultraviolet (UV) laser illumination. Using this technique, the authors immobilized RGD adhesion peptide, a recombinant fibronectin fragment FN_9–10_, and platelet‐derived growth factor B (PDGF‐BB) within defined regions of separate hydrogels embedded with hMSC microtissues. hMSCs invaded significantly more into the regions with the patterned factors, demonstrating the ability to control stem cell behavior in three‐dimensions through the scaffold system (Figure 3B).

Hydrogels can also be designed to respond to two‐photon excitation using near‐infrared (NIR) light,^[^ ^31^ ^]^ enabling complex 3D pattern generation via a NIR laser which generates a limited excitation volume. Using a PEG hydrogel crosslinked with allyl sulfide groups in the presence of photoinitiator, Gandavarapu et al. demonstrated two‐photon patterned attachment, and subsequent patterned exchange of ligands in the 3D volume.^[^ ^32^ ^]^ The authors used this technique to form an RGD peptide pattern, demonstrate hMSC attachment to the patterned region, and perform subsequent ligand exchange in the presence of seeded cells with no adverse effects. Wylie et al. demonstrated two‐photon mediated patterning of multiple growth factors simultaneously within 3D hydrogels.^[^ ^33^ ^]^ An agarose hydrogel containing coumarin photocaged thiols was photoactivated and reacted with one maleimide‐conjugated linker, followed by a second distinct maleimide‐conjugate to allow individually controlled immobilization of two stem cell differentiation factors. Using this technique, the authors demonstrated precisely controlled 3D patterning of sonic hedgehog (SHH) and ciliary neurotrophic factor (CNTF), and showed bioactive responses within retinal precursor cells. This system was additionally used to induce migration of neural precursor cells into the gel depth along a photodefined SHH gradient.

Photoresponsive scaffolds can also be utilized to control the timing of bioactive cue presentation. Kloxin et al. designed a hydrogel platform for time‐controlled presentation of adhesive peptides as well as photocontrolled degradation.^[^ ^34^ ^]^ The authors incorporated RGDS adhesion peptide linked via a nitrobenzyl ether‐derived photolabile tether into PEG‐based hydrogel scaffolds. Upon application of 365 nm light, the RGDS tether was photocleaved, allowing modulation of the timing of RGDS ligand presentation to encapsulated hMSCs. Photocleavage of RGDS from the scaffold on day 10 of culture increased glycosaminoglycan (GAG) production and induced differentiation of hMSCs down a chondrogenic pathway, while maintaining their viability in culture. This timecourse of ligand presentation approximates the native course of hMSCs differentiating into chondrocytes whereby hMSCs produce the adhesion protein fibronectin (with RGDS epitopes) in their initial phase, with downregulation of fibronectin between days seven and ten. Azagarsamy et al. utilized a multiwavelength approach to control sequentially‐timed release of multiple growth factors within a hydrogel.^[^ ^35^ ^]^ Conjugating proteins to a PEG hydrogel using two distinct photocleavable moieties (nitrobenzyl and coumarin methylester) allowed individually timed and tunable release of each payload via 405 nm and 365 nm light, respectively. This system was used to sequentially deliver bone morphogenetic protein (BMP)‐2 (on day 1) and BMP‐7 (on day 4) to hMSC‐laden gels and resulted in increased alkaline phosphatase (ALP) (osteogenic) activity due to the temporally controlled protein exposure.

**Figure 4:**
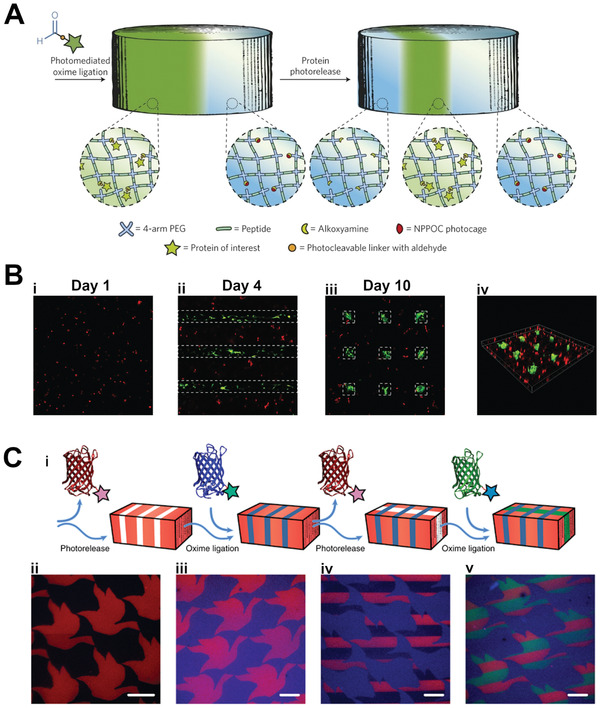
Photocontrolled biochemical cue presentation: reversible 3D/4D patterning. A) Schematic of reversible photopatterning strategy where proteins functionalized with a photocleavable linker are tethered to the gel via photomediated oxime ligation allowing subsequent photopatterned removal. B) Confocal fluorescence images of hydrogels with hMSC (red) imaged on i) day 1 before photomediated vitronectin patterning, on ii) day 4 (after 3 days of vitronectin patterning) where cells show osteocalcin (green) staining in the patterned regions (dashed rectangles), and iii,iv) day 10 (6 days after selective photomediated removal of vitronectin) showing osteocalcin only in the vitronectin‐functionalized islands (dashed rectangles). Adapted with permission.^[^ ^36^ ^]^ Copyright 2015, Springer Nature. C‐i) Schematic of 4D photopatterning strategy where photorelease of fluorescent sortagged proteins can be combined with photomediated oxime ligation of distinct tagged proteins to create complex patterns over time. Confocal fluorescent images of patterns in hydrogels (initially uniformly functionalized with mCherry‐*o*NB‐N_3_, red) created by ii) photorelease of mCherry (and uncaging of sites for subsequent ligation), iii) photomediated oxime ligation of mCerulean‐CHO (blue), iv) additional photorelease of mCherry leaving the mCerulean pattern intact, and v) oxime ligation of EGFP‐*o*NB‐CHO (green, EGFP = enhanced green fluorescent protein). Scale bars, 100 µm. Adapted with permission.^[^ ^20^ ^]^ Copyright 2019, Springer Nature.

Further control of stem cell manipulation can be afforded through the development of photocontrolled scaffolds which allow both the introduction and subsequent removal of biological cues. DeForest and Tirrell developed a PEG‐hydrogel‐based system incorporating photoresponsive linkers which allowed for both ligation and subsequent scission.^[^ ^36^ ^]^ Using a sequence of photodeprotection‐oxime‐ligation (with 2‐(2‐nitrophenyl)propyloxycarbonyl (NPPOC) as the photocaging group) enabled the controlled 3D patterning of functionalized proteins into the hydrogel, while an *ortho*‐nitrobenzyl ester (*o*NB) photoscission reaction allowed subsequent 3D‐patterned removal (**Figure** **4**A). This system was used to control the photoreversible patterning of vitronectin within scaffolds with encapsulated hMSCs. Cell‐seeded gels were first labeled in bulk with vitronectin after one day of culture, which was then uniformly photocleaved three days later. Encapsulated hMSCs showed increased osteocalcin (OCN) staining and ALP activity (indicative of osteogenesis) by day 4 (after introduction of the vitronectin), and these levels returned to baseline after removal of the cue. This time course of introduction and removal was also performed on gels where the vitronectin was patterned into distinct volumes within the gel and then subsequently removed from additional subvolumes. hMSC osteogenesis was seen in the vitronectin‐patterned volumes by day 4, but by day 10 was maintained only in the regions where vitronectin was not subsequently removed, thus demonstrating this system as a platform for reversible 3D‐patterned stem cell differentiation (Figure 4B).

Recent advances in protein modification techniques have expanded the capabilities of photocontrolled protein presentation within scaffolds, allowing for precise tethering of fragile proteins while retaining bioactivity. Shadish et al. used a sortase‐mediated transpeptidation approach to create a library of proteins with bioorthogonal reactive handles, allowing for their bioactive and reversible patterning within PEG hydrogels.^[^ ^20^ ^]^ Using this technique, the authors demonstrated complex photocontrolled pattern evolution over time, involving the photomediated addition and removal of multiple proteins in the same material (Figure 4C). Dynamic patterning of epidermal growth factor (EGF) was shown to influence distribution of A431 squamous carcinoma cells across the gel and temporally control EGF internalization on the subcellular level. In an additional work, Shadish et al. demonstrate a genetically encoded system for photocontrolled patterned protein release from hydrogel scaffolds.^[^ ^37^ ^]^ This versatile approach allows the creation of bioactive fusion proteins, with the protein of interest linked to the hydrogel scaffold via a visible light (400 nm) photocleavable protein, PhoCl. These techniques have potential for future use for spatiotemporal control of stem cell lineage with precise control of multiple growth factor presentation within a cell‐seeded hydrogel matrix.

While the previous techniques have involved the controlled presentation of specific cues for cell interaction, photoresponsive scaffold strategies have also been investigated for their potential to control cell fate by modulating nonspecific protein interactions. Bai et al. developed a photoresponsive zwitterionic hydrogel utilizing the photoinduced chemical changes of an incorporated spiropyran photoisomer.^[^ ^38^ ^]^ By tuning the individual strengths of constructively interacting NIR and green light, the authors were able to reversibly control the balance between hydrophobic and hydrophilic moieties within the gel and modulate nonspecific interactions between hMSCs and the culture platform. This platform allowed photocontrol of hMSC differentiation toward a more adipogenic or osteogenic fate, depending on the applied light wavelength when cells were cultured in bipotential differentiation medium. Temporal control of the wavelength of illumination was also used to suspend the differentiation of encapsulated cells at user‐defined time points. Through the use of a photomask, the authors were additionally able to pattern hMSC differentiation behavior to constrained spatial regions of the hydrogel. Photoisomer material‐based techniques could present an intriguing alternative strategy for influencing stem cell lineage.

### 2.2 Photoinduced Mechanical Changes

Cells sense the mechanical properties presented by the local matrix environment and respond to these mechanical cues.^[^ ^39^, ^40^, ^41^ ^]^ The physical forces encountered can influence the behavior and biological fate of the cell, including stem cell lineage commitment, migration, and motility.^[^ ^42^, ^43^ ^]^ The process by which cells sense these physical forces and transduce them into biological responses has been described in several extensive reviews on mechanotransduction.^[^ ^42^, ^44^, ^45^ ^]^ Synthetic gels allow for the independent control of ligand presentation and mechanical properties, enabling isolation of these parameters.^[^ ^43^ ^]^ A number of studies have used photomediated techniques to manufacture a substrate of patterned elasticity for subsequent cell seeding and investigation of stem cell response.^[^ ^46^, ^47^ ^]^ Here we highlight materials which can be used for externally mediated in situ manipulation of seeded cells using an applied photostimulus.

**Figure 5:**
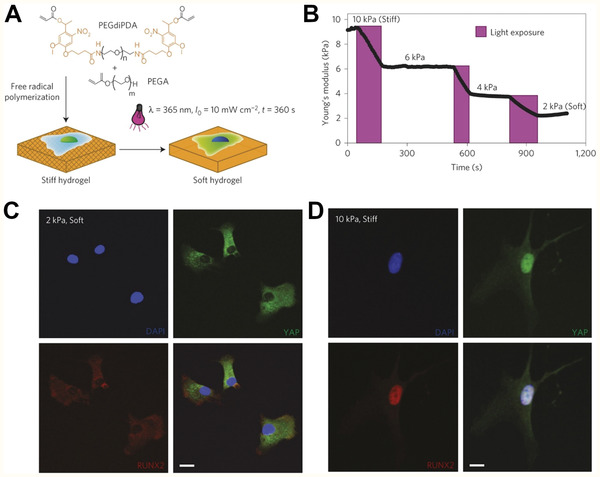
Photoinduced changes of substrate mechanical properties. A) Hydrogels consisting of photodegradable PEGdiPDA crosslinker and monoacrylated PEG (PEGA) form a stiff (≈10 kPa) hydrogel which can be photodegraded to ≈2 kPa upon 365 nm light exposure. B) Defined doses of light exposure can generate hydrogels with a range of moduli from stiff to soft. C) hMSC cultured on soft hydrogels (2 kPa) show YAP (green) and RUNX2 (red) excluded from the nucleus (blue), indicating deactivation. D) hMSC on stiff gels (10 kPa) show nuclear‐localized (activated) YAP and RUNX2. Scale bars, 20 µm. Adapted with permission.^[^ ^50^ ^]^ Copyright 2014, Springer Nature.

As stem cell differentiation can be highly influenced by substrate stiffness,^[^ ^44^, ^48^, ^49^ ^]^ photoresponsive gels that allow for manipulation of the material modulus of elasticity can be used to investigate the temporal characteristics of this process. Such materials can be utilized to investigate what “mechanical dose” leads to irreversible activation of a mechanotransduction pathway and under what conditions this activation is reversible. The ability to change the stiffness of the substrate without causing other disturbance to the cells is critical to prevent the introduction of possible confounding factors. Yang et al. developed a method to change the stiffness of the substrate by using a photodegradable PEG‐based gel formed by free‐radical polymerization of a nitrobenzyl ether‐containing photodegradable crosslinker, PEGdiPDA, and a monoacrylated PEG (**Figure** **5**A).^[^ ^50^ ^]^ Exposure to 365 nm light caused photocleavage of the crosslinker and subsequent softening of the gel (Figure 5B). This allowed hMSC seeded on the gel to be “mechanically dosed” with different stiffness conditions for controlled durations. Growth on a stiff gel caused activation of the preosteogenic transcription factor RUNX2, Yes‐associated protein (YAP), and transcriptional coactivator with PDZ‐binding domain (TAZ) (Figure 5C,D). The activation of these pathways could be reversed by softening the gel substrate via light exposure. Above a certain threshold of temporal exposure to the stiff substrate these pathways became irreversible indicating that the hMSCs have a mechanical memory of past exposure to stiff substrates.

Another method to phototunably modulate hydrogel stiffness was demonstrated by Killaars et al. through the use of a copolymerizing eight‐arm PEG thiol with a maleimide allyl sulfide crosslinker.^[^ ^51^ ^]^ The stiffness of these gels could be softened through a radical‐mediated addition‐fragmentation chain transfer (AFCT) process created in the presence of the photoinitiator lithium phenyl‐2,4,6‐trimethylbenzoylphosphinate (LAP) and glutathione with exposure to 365 nm light. This fragmentation of the gel and subsequent softening had measurable effects on hMSC cultured on the gel substrate. The authors found that histone acetylation and chromatin organization changed quickly in response to gel substrate softening. These changes were reversible or irreversible depending on the duration of cell exposure to the stiff substrate prior to photosoftening. These findings suggest that epigenetic remodeling might be involved in “memory‐keeping” of the stiffness exposure history of the cell.

As cell morphology can also be impacted by substrate stiffness,^[^ ^3^, ^52^, ^53^ ^]^ dynamically changing the substrate stiffness while cells are attached can affect morphology and differentiation outcomes. Guvendiren et al. demonstrated this by developing a sequential crosslinking technique using a modified methacrylated hyaluronic acid (MeHA) that can be crosslinked when exposed to thiols or free radicals.^[^ ^54^ ^]^ The polymers were first cured into a gel using the thiol reaction by introducing dithiothreitol (DTT). The DTT concentration was chosen so that not all of the methacrylates were consumed in this initial gelation. The gel was subsequently stiffened at a specifically chosen time by reacting the remaining methacrylates through the introduction of a photoinitiator (Irgacure 2959) and exposure to UV light to create free radicals. The authors showed hMSC seeded onto the surface of these gels reacted to the increase in substrate stiffness within hours with increased cell area, motility and traction forces. When cultured in bipotential media over time spans of 14 days, the hMSCs that experienced increasing substrate stiffness earlier in culture favored osteogenic differentiation whereas increasing stiffness later in culture favored adipogenic differentiation.

Material systems can also be designed to permit wavelength‐dependent control of mechanical properties, allowing for additional complex manipulations. Kalayci et al. demonstrated the ability to initiate gelation and then increase the stiffness of this gel on‐demand.^[^ ^55^ ^]^ This system used a PEG based hydrogel with a [2 + 2] cycloaddition reaction of styrylpyrene (SP) and acrylamidylpyrene (AP) which have different crosslinking properties at 455 and 420 nm. A 4arm‐SP‐AP‐PEG polymer showed gelation when exposed to 455 nm light and increased its stiffness when subsequently exposed to 420 nm light. The authors observed shrinkage of the gel after the 420 nm light exposure and used this shrinkage to detach a 2D culture of human foreskin fibroblasts from the surface of gel, opening the way for applications in cell sheet engineering. They also demonstrated viability of hMSC at 24 h postencapsulation, indicating promise for future application with stem cell manipulation. Another wavelength‐selective system designed by Truong et al. featured a PEG‐based hydrogel that allowed for a wavelength‐gated reversible photochemical reaction to crosslink and then decouple the polymers thereby enabling reversible modification of mechanical properties.^[^ ^56^ ^]^ They used a reversible [2 + 2] cycloaddition of methoxy‐PEG‐styrylpyrene where exposure to 400–500 nm light resulted in crosslinking which was reversed when exposed to 340 nm light. The authors demonstrated that several cycles of reversible crosslinking were possible in the same gel material and that a fractured gel exposed to 340 nm light followed by 400–500 nm light could reanneal and allow for hydrogel “self‐healing.” hMSC were encapsulated within the photoexposed gel with high viability over 24 h. Multiwavelength strategies show promise as an elegant tool for future studies of stem cell fate and memory on matrices with dynamically tunable mechanical environments.

Photoresponsive systems can also be used to isolate and study the effect of individual parameters on cell fate. The ability of cells to degrade their surrounding matrix when encapsulated within a hydrogel can affect their behavior and differentiation. Khetan et al. investigated this by using a hyaluronic acid (HA)‐based gel functionalized with both maleimide and methacrylate groups.^[^ ^57^ ^]^ The maleimide functionalization allowed crosslinking with matrix metalloproteinase (MMP)‐degradable peptide linkages which could be degraded by encapsulated hMSCs. The gels were also incubated with photoinitiator (Irgacure 2959) so that exposure to UV light would create methacrylate crosslinking which the cells could not degrade, thus creating a more restrictive gel. The concentration of monomers was adjusted so that both restrictive and less restrictive gels had equivalent elastic moduli. Gels that were less restrictive showed more cell‐mediated degradation, higher levels of cell spreading and traction, and led to osteogenesis. Gels that were more restrictive resulted in less overall cell‐mediated degradation and showed less cell spreading and less traction, and led to adipogenesis. Delayed UV‐mediated secondary crosslinking to the more restrictive state was also shown to cause a switch from osteogenesis toward adipogenesis without altering cell shape. The photoresponsive platform allowed the authors to decouple the parameters of cell‐spreading and hydrogel degradation to study the influence of matrix degradation on cell fate decision.

### 2.3 Photodefined Topographies

**Figure 6:**
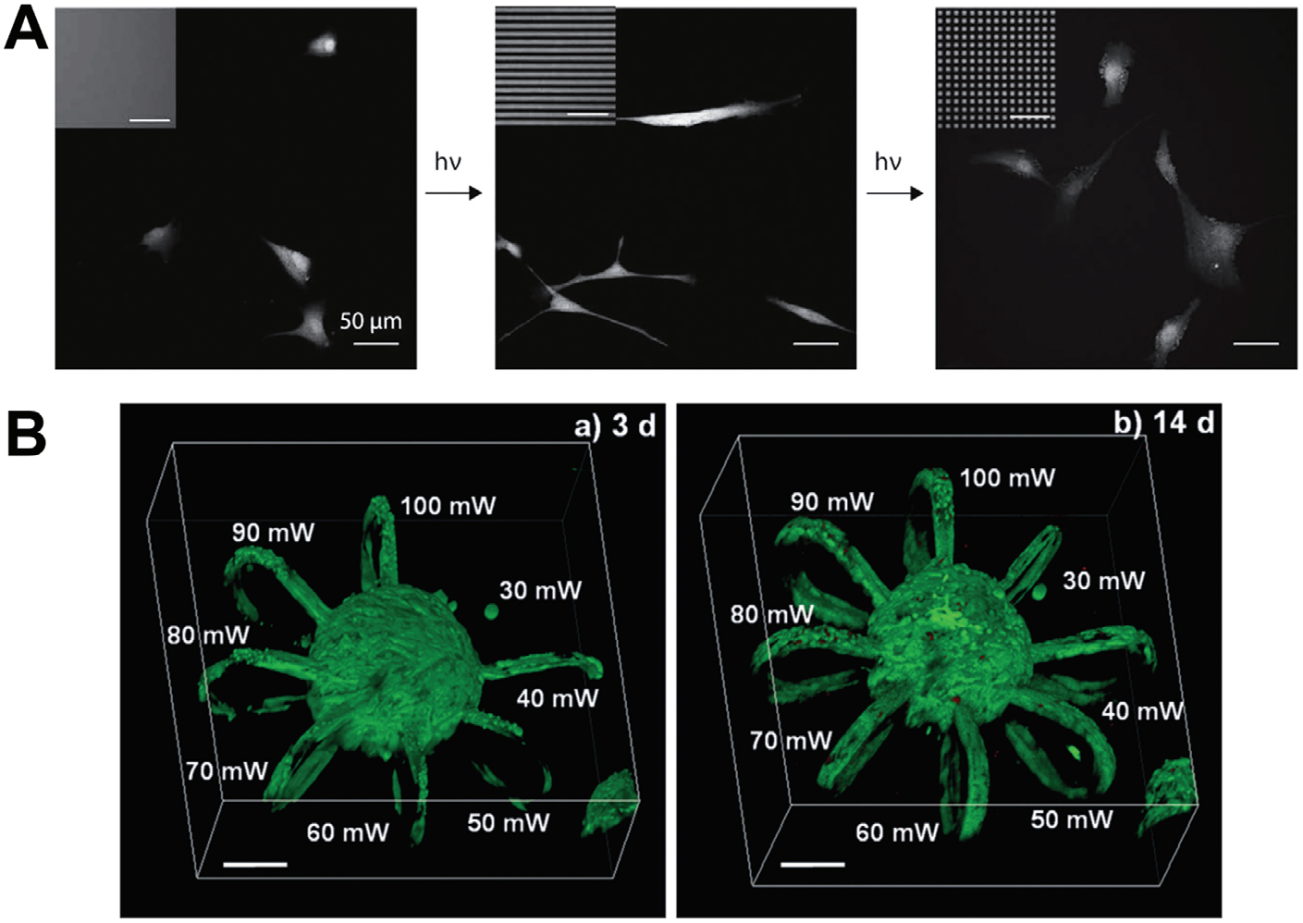
Photodefined topographies. A) Smooth hydrogel substrates (left) are sequentially photopatterned to form anisotropic channels (center) and isotropic squares (right). hMSC seeded on smooth substrates showed a rounded morphology, then acquired an elongated shape along the feature axis of the patterned channels, and finally reacquired a rounded shape on the squares. Reproduced with permission.^[^ ^58^ ^]^ Copyright 2013, Wiley‐VCH. B) Green fluorescent protein (GFP)‐labeled hTERT‐immortalized human adipose‐derived mesenchymal stem cells, encapsulated within hydrogel as spheroids, spread into photodegraded 3D microloop channels formed using two‐photon excitation at varying laser powers. At 3 days, cells had spread into channels fabricated at laser powers down to 40 mW and by 14 days cells had entered the 30 mW eroded channel. Scale bars, 100 µm. Reproduced under the terms of the Creative Commons Attribution 4.0 International Public license.^[^ ^59^ ^]^ Copyright 2018, the Authors. Published by Wiley‐VCH GmbH.

Photoresponsive materials can also be used to create defined topographies for controlling stem cell behavior. Selective photodegradation techniques can generate topographical features on a hydrogel surface via controlled hydrogel erosion using a photomask. Kirschner and Anseth employed this approach to create features with subcellular dimensions and varying aspect ratios using a photolabile PEG hydrogel system which also allowed for dynamic in situ feature alteration.^[^ ^58^ ^]^ hMSC showed increased elongation and alignment in response to higher aspect ratio static features and additionally showed reversible morphology changes in response to sequentially presented topographies (**Figure** **6**A). 3D topographies can also be defined within the bulk of photomanipulatable scaffolds by using a focused laser beam. Lunzer et al. presented a two‐photon 3D degradative approach, encapsulating immortalized human adipose‐derived mesenchymal stem cells within HA‐based hydrogels with photocleavable *ortho*‐nitrobenzyl ester PEG linkages.^[^ ^59^ ^]^ The low absorption cross sections of photoresponsive moieties such as *ortho*‐nitrobenzyl derivatives can present a challenge for two‐photon based techniques, requiring increased laser intensities and irradiation times which may compromise efficiency and/or cell viability. Lunzer et al. addressed this efficiency challenge through inclusion of a water‐soluble photosensitizer in their system. Two‐photon excitation was used to generate 3D microloop architectures within the hydrogel bulk and cells were able to spread into these features, creating complex 3D cell patterns at moderate laser power (Figure 6B).

### 2.4 Photoinduced Stem Cell Release

Photoresponsive scaffolds can also be designed to allow controlled release of stem cells from the scaffold for further use, analysis, or manipulation. You et al. demonstrated a platform for phototriggered release of mouse embryonic stem cell (mESC) colonies from the surface of heparin‐based hydrogels.^[^ ^60^ ^]^ The authors were motivated by a need to recover specific groups of differentiating stem cells grown on the surface of growth factor‐loaded heparin gels to allow for genetic analysis at specific time points without disturbance to surrounding cells. The photoresponsive gels were formed by polymerizing a photocleavable crosslinker containing *ortho*‐nitrobenzyl groups and thiolated heparin (Hep‐SH) using a free radical thiol‐ene coupling reaction that was chemically initiated. Exposure to 365 nm light caused degradation of the gel only in the exposed region. mESC adhesion to the surface of the gel was accomplished by printing fibronectin onto the gel surface. This allowed colonies of cells to grow in localized areas of the gel while maintaining the same growth factor exposure. Light exposure degraded the gel under individually selected colonies allowing them to dissociate from the bulk gel with minimal disturbance to the collected cells or their neighboring colonies. The mESCs retrieved in this manner maintained their proliferative abilities and were shown to differentiate into endoderm through room temperature real‐time polymerase chain reaction (RT‐PCR) analysis of pluripotency.

Photocontrolled disassembly of hydrogel components can allow stem cell release as well as the controlled release of protein payloads. Wang et al. demonstrated this by designing a unique light‐sensing protein‐based hydrogel comprised of photoreceptor C‐terminal adenosylcobalamin binding domain (CarH_C_) protein polymers.^[^ ^61^ ^]^ These CarH_c_ polymers were formed using the genetically encoded SpyTag‐SpyCatcher system for protein conjugation. Adenosylcobalamin (AdoB_12_)‐induced CarH_C_ tetramerization resulted in gelation of the polymers, forming an elastic hydrogel. The result was a protein‐polymer that self‐assembled in the dark in the presence of AdoB_12_ and rapidly disassembled in the presence of 522 nm or white light. The authors showed successful encapsulation of 3T3 fibroblasts and hMSCs into these gels and release of the cells upon light exposure, with high cell viability. They additionally demonstrated controlled release of protein payloads from the gels by incorporating an mCherry‐CarH_c_ fusion into the hydrogel network which was released from the gel upon light irradiation.

Photostimulation can be combined with other chemical stimuli to engineer precise control of hydrogel degradation to control cell release. Badeau et al. demonstrated that by linking different types of triggerable linkers together they could create a Boolean logic‐based control system for hydrogel degradation.^[^ ^62^ ^]^ They established YES/OR/AND control using MMP‐8 for enzymatic cleavage of a proteolytically sensitive peptide sequence, tris(2‐carboxyethyl)phosphine (TCEP) as a reductive trigger for disulfide bonds, and an *ortho*‐nitrobenzyl ester group for light activation. YES gates were created by one stimuli sensitive moiety in the crosslinker, whereas OR gates contained two stimuli‐labile units in series. The AND gate was created by using a cyclic architecture where two unique stimuli were required to trigger protein release. The authors created 17 different four‐arm PEG‐tetrabicyclononyne (PEG‐tetraBCN) based materials that displayed all of the possible YES/OR/AND logic outputs using the three inputs as described above. This system was used to demonstrate logic‐based gel release of the chemotherapeutic doxorubicin. The authors also demonstrated the release of embedded hS5 bone marrow‐derived stromal cells using different combinations of stimuli to create distinct spatial release patterns within the same gel. Notably, this technique has also been extended to create stimuli‐responsive logic gate‐based linkers to control the release of site‐specifically modified proteins, by inserting these singular or multistimuli responsive linkages between the protein and its gel tether. Gawade et al. demonstrated the use of the YES/OR/AND Boolean logic degradation system to show the successful release of fluorescent proteins from the gels in different temporal patterns based on the combination of the linkers and the order of the trigger presentations.^[^ ^63^ ^]^ The authors additionally demonstrated spatial pattering with the use of masked light exposure. This sets the stage to be able to precisely guide embedded stem cells through differentiation programs by creating different spatial and temporal patterns of protein cues.

### 2.5 In Vivo and Organoid Applications

Photoactivated scaffolds are a powerful tool for studying stem cell response to precisely‐controlled environmental stimuli. As photoresponsive systems continue to evolve to create more complex architectures and patterns of spatiotemporal cues, careful design and thorough characterization should be applied to ensure that the materials and applied stimuli are biocompatible over extended time periods and free of toxic side effects that could confound observed cellular responses.

In vivo stem cell manipulation via photocontrolled scaffolds remains an area of continued interest. Lee et al. promoted endogenous cell adhesion and vascularization through in vivo photomanipulation of PEG‐based scaffolds implanted in a mouse model by activating photocaged RGD peptides.^[^ ^64^ ^]^ The subcutaneously implanted hydrogels were activated by transdermal light exposure (351 nm, 20 mW cm^−2^, 10 min). The authors were additionally able to pattern the area of RGD activation using a photomask to limit light exposure to particular regions of the skin and underlying hydrogel. Stowers et al. demonstrated dynamic in vivo modulation of hydrogel stiffness in a mouse model by phototriggering calcium release from gold nanorod‐loaded liposomes within an alginate hydrogel, increasing crosslinking density.^[^ ^65^ ^]^ NIR light applied transdermally (808 nm, 1.78 W cm^−2^, 5 min) to subcutaneously implanted alginate/liposome samples in a mouse model increased the storage moduli of the hydrogels. These techniques could pave the way for in vivo photomanipulation of implanted stem cell scaffolds. As photoresponsive scaffolds are developed toward potential in vivo clinical use, important considerations will include cost and thorough investigation of the in vivo safety profile of the material system including byproducts of photoconjugation and photocleavage. Tissue depth penetration of a transdermally applied photostimulus is also an important consideration^[^ ^66^ ^]^ which may engender the use of NIR‐activated approaches^[^ ^22^ ^]^ and limit the capability of patterning high‐resolution features due to light scattering. Yet, considering that photoactivated therapies have reached clinical approval,^[^ ^67^ ^]^ photoactivated scaffolds for stem cell manipulation represent a promising avenue for in vivo application.

Another exciting area of potential application for photocontrolled scaffolds lies in guiding organoid development.^[^ ^68^, ^69^, ^70^ ^]^ Through modification of the matrix bioactive cue presentation or physical topography, materials can be designed to guide multipotent cells toward the formation of in vivo‐like heterogeneous cell type orientations and functions.^[^ ^71^ ^]^ A photostimulus could be used as the trigger for “breaking symmetry” of initially homogeneous stem cell aggregates to move toward specified lineages and multicellular formations.^[^ ^68^ ^]^ Recent work by Hushka and co‐workers utilized a hydrogel capable of phototunable matrix softening to direct intestinal organoid crypt size and formation.^[^ ^72^ ^]^ Photocontrolled scaffolds also hold potential to guide tissue morphogenesis via 3D patterning of growth factors. Broguiere et al. recently demonstrated a two‐photon patterning technique to guide axons within a hyaluronan‐based matrix via photopatterned nerve growth factor.^[^ ^73^ ^]^ Photoresponsive platforms designed for controlled organoid development and tissue morphogenesis could serve as valuable in vitro models for studying early‐stage tissue development as well as understanding disease progression and studying the efficacy of therapeutic interventions at specific disease stages.

Taken together, photoresponsive materials hold significant potential for future application in guiding stem cell response for both in vivo therapies and in vitro recapitulation of tissue‐like structure and function.

## 3 Electrical Stimulation

**Figure 7:**
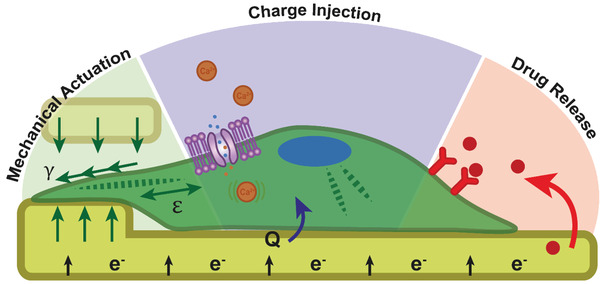
Schematic of an electrically responsive biomaterial stimulating a cell. Mechanical actuation acts physically upon the cell, inducing stress or strain intracellularly and/or shear flow over the cell externally. Charge injection (*Q*) from the biomaterial to the cell has been demonstrated to stimulate intracellular Ca^2+^, increase calcium deposition, influence voltage gated ion channels, and affect the actin cytoskeleton. Drug release can also be controlled via loading molecules into electrically responsive materials to release upon an electrical signal.

Many tissues within the body generate endogenous electric cues which are critical for a variety of biological mechanisms, such as angiogenesis, mitosis, cell signaling, migration, and wound healing. In tissue engineering, appropriating biophysical cues from the in vivo microenvironment is an important approach for enhancing targeted cellular response. Hence in turn, the application of exogenous electrical signals has been observed to influence stem cell differentiation,^[^ ^13^ ^]^ leading to a field of research focused on using electrical stimulation as a tool to guide and control stem cell fate. There is a large body of work focusing on the application of electrical fields for stem cell control wherein an external electrical field is applied to the cell culture, either capacitively coupled or an external electromagnetic field, which has been discussed thoroughly in other reviews.^[^ ^74^ ^]^ Furthermore, the use of conductive biomaterials in bioelectronics for stimulating electrodes is extensive, particularly in the neural interfacing areas and with neural stem cells.^[^ ^12^, ^75^ ^]^ The stimulation of cells here is focused specifically on multipotent stem cell response to an electrical signal, and how this type of external stimuli can drive stem cell differentiation and migration. Primarily electrical stimulation is transduced into three main responses from an electrically responsive biomaterial scaffold; charge injection into the adherent cell, drug or molecular release from the scaffold, and mechanical actuation acting upon the cell (**Figure** **7**).

### 3.1 Conductive Biomaterials

**Figure 8:**
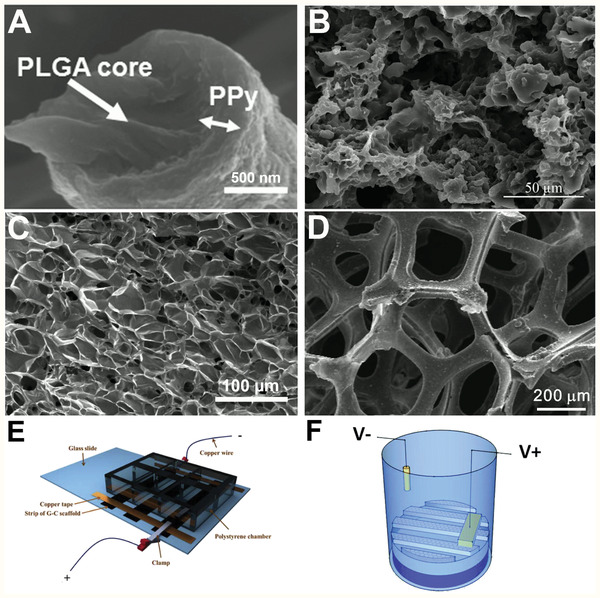
Scanning electron microscopy (SEM) micrographs of conductive polymer biomaterials scaffolds. A) Polypyrrole coated PLGA fibers. Reproduced with permission.^[^ ^84^ ^]^ Copyright 2016, Wiley‐VCH GmbH. B) Polyaniline/PCL scaffold. Reproduced with permission.^[^ ^88^ ^]^ Copyright 2015, Wiley‐VCH GmbH. C) PEDOT:PSS hydrogel. Reproduced with permission.^[^ ^96^ ^]^ Copyright 2018, Springer Nature. D) 3D‐graphene foam. Reproduced with permission.^[^ ^97^ ^]^ Copyright 2013, Springer Nature. Graphical schematics of A) substrate mediated electrical stimulation cell culture set up. Reproduced with permission.^[^ ^98^ ^]^ Copyright 2019, Elsevier. B) Charge injection cell culture set up where the conductive biomaterial is the working electrode, and an anode is submerged into the culture media. Reproduced with permission.^[^ ^99^ ^]^ Copyright 2016, The Royal Society of Chemistry.

Conductive biomaterials are typically synthesized from intrinsically conductive materials which are biocompatible, or conductive components incorporated into a biocompatible matrix. One of the most common intrinsically conductive biomaterials used to interface with cells are conductive polymers.^[^ ^76^ ^]^ These include polypyrrole (PPy),^[^ ^77^ ^]^ polyaniline (PANI),^[^ ^78^ ^]^ and polythiophene derivatives, commonly poly(3,4‐ethylenedioxythiophene) polystyrene sulfonate (PEDOT:PSS).^[^ ^79^ ^]^ This class of materials are widely used as biomaterials and in tissue engineering applications, with many advantageous properties compared to more conventional conductive materials (i.e., metals or metal oxides). Conductive polymers have very good conductivity, and they are easy to synthesize and tailor according to need. These polymers’ properties can be tailored to change various important properties such as stiffness, roughness, and surface chemistry, and can be modified to incorporate biological factors to enhance cellular adhesion and interaction.^[^ ^80^, ^81^, ^82^, ^83^ ^]^ Typically they are synthesized through electrochemical polymerization or vapor phase deposition, hence a conformal film can be grown on a conductive substrate or deposited onto a surface coated with an oxidant. Furthermore, there are a variety of techniques to create scaffolds and structures with these materials; conductive polymers are often combined with electrospinning^[^ ^84^, ^85^, ^86^ ^]^ or templating synthesis techniques to create fibrous or porous conductive polymer scaffolds (**Figure** **8**A,B).^[^ ^87^, ^88^ ^]^ While these polymers have advantageous properties, they can also be brittle and nondegradable which can be problematic for long‐term tissue engineering applications. These issues can be mediated through incorporating the conductive polymers into coblock polymers or conjugated as oligomers to graft onto other polymer systems.^[^ ^89^, ^90^, ^91^, ^92^ ^]^ There is also a large body of work exploring the use of conductive polymers as hydrogel composite materials for biomaterial scaffolds (Figure 8C).^[^ ^93^, ^94^, ^95^ ^]^

Conductive carbon allotropes such as graphene, graphene oxide, and carbon nanotubes are another popular choice when creating conductive biomaterials.^[^ ^100^, ^101^ ^]^ Their excellent conductivity, large‐scale and low‐cost production, and processability are advantageous factors. These materials can be used in cell culture as thin films and substrates,^[^ ^102^, ^103^ ^]^ and have been used to create standalone biocompatible 3D scaffolds.^[^ ^97^ ^]^ These materials can also be fragile and difficult to handle when creating cell culture set‐ups to deliver electrical stimulation, and furthermore are often prepared as films on glass or other very hard substrates. As stiffness is a well‐known and greatly influential factor in stem cell differentiation,^[^ ^3^ ^]^ creating composite materials which combine the conductivity of these carbon materials with supportive materials which have more physiologically‐relevant moduli is a promising area of research. This can involve coating a nonconductive substrate with a layer of the conductive material; this is an advantageous approach as if the layer of conductive material is thin enough the underlying substrate stiffness sensed by cells can be adopted,^[^ ^104^ ^]^ or even conform to topographical features^[^ ^99^ ^]^ while also introducing conductivity to stimulate cells. For a scaffold, the encapsulating or composite material is usually a nonconductive biocompatible matrix, typically a hydrogel or polymer, and both synthetic and natural materials are commonly used.^[^ ^105^, ^106^, ^107^, ^108^ ^]^ Similarly to the conductive polymers, these materials can be processed into structured scaffolds through electrospinning or template synthesis (Figure 8D).^[^ ^109^ ^]^

### 3.2 Conductivity Mechanisms

Conductive biomaterials are typically used to conduct an electrical signal and interact with cells through two routes: substrate‐mediated electrical field, or charge injection (Figure 8E,F). In the first approach, a conductive biomaterial is connected in series into an electrical circuit and an electrical signal is passed through the biomaterial. This will induce a local electrical field and charge is not transferred from the biomaterial to the surrounding electrolyte. The second approach uses the conductive biomaterial as a “working electrode” and an opposing inert counter electrode to facilitate charge transfer through the surrounding electrolyte (i.e., cell media), thus facilitating charge injection from the biomaterial to the interfacing cells. Depending on the nature of the biomaterial, this charge injection can be faradaic or capacitive.^[^ ^110^ ^]^ A faradaic charge injection involves electrons being transferred between the biomaterial and the cell, resulting in a redox reaction which occurs at the cell‐biomaterial interface. This type of charge injection can be problematic as new chemical species are generated at the biomaterial‐tissue interface, and possibly irreversible chemical reactions such as water electrolysis, changes in the local pH, or protein reduction/oxidation. A capacitive injection mechanism occurs when the charge is transferred through charged chemical species via a double‐layer at the cell–biomaterial interface. This charge injection mechanism is dictated by the biomaterial properties; for example, conductive carbon materials such as graphene or carbon nanotubes inject charge in a capacitive manner while metal oxides such as iridium oxide (commonly used in neural electrodes) have a faradaic charge injection due to reversible reactions between the Ir^3+^ and Ir^4+^ states. Conductive polymers readily conduct an electrical charge via the conjugated polymer backbone in the presence of a dopant molecule (typically anionic), and undergo reversible oxidation/reduction when a potential is applied. Hence they can be described as “pseudocapacitive” as they inject charge via movement of charged chemical species as the polymer dopes or de‐dopes with available electrolytes without undergoing a chemical reaction. The efficiency and capability of a material to deliver a biologically safe electrical signal is dictated by the charge injection limit; the charge injection limit is commonly defined as the maximum charge that can be delivered before water electrolysis occurs.^[^ ^111^ ^]^ When designing conductive biomaterials to safely stimulate cells, these are important factors to consider.

### 3.3 Cellular Response to Electrical Stimulation

**Figure 9:**
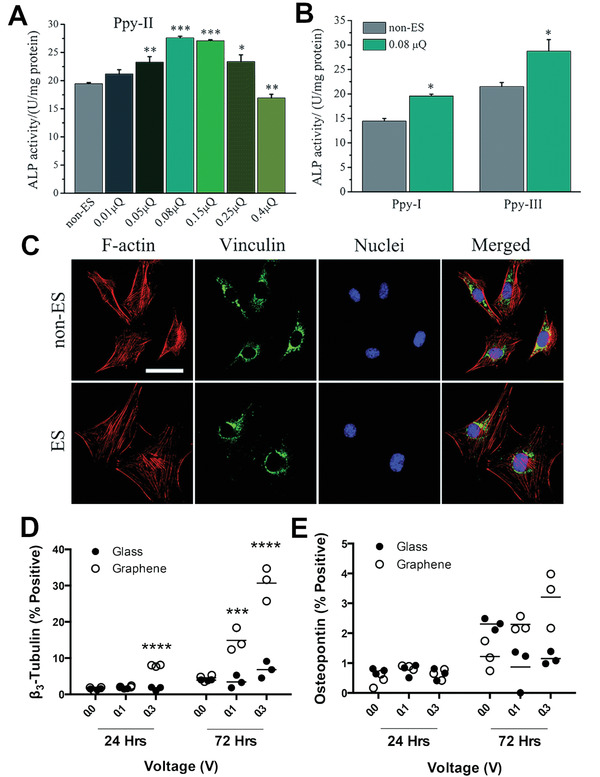
A) ALP activity on PPy‐II electrodes with different *Q*_inj_ and B) ALP activity on PPy‐I and PPy‐III electrodes with *Q*_inj_ of 0.08 µC. ^*^ *p* < 0.05, ^**^ *p* < 0.01, ^***^ *p* < 0.001. C) Cellular morphologies on the Ppy‐II were indicated by immunofluorescent staining after 2 days of stimulation (0.08 µQ, 5 mV). Cells were stained for the focal adhesion protein vinculin (green), cellular nuclei (blue), and actin cytoskeleton (red). The images share the same scale bar of 50 µm. Reproduced with permission.^[^ ^114^ ^]^ Copyright 2018, The Royal Society of Chemistry. Flow cytometry‐based quantification of stimulated and unstimulated hMSCs expressing D) *β*3‐tubulin, and E) osteopontin in hMSCs grown on glass, unpatterned graphene, and patterned graphene. Adapted with permission.^[^ ^99^ ^]^ Copyright 2016, The Royal Society of Chemistry.

Direct electrical stimulation, including charge injection and substrate‐mediated electrical stimulation, has been observed to influence stem cell behavior on conductive biomaterial planar substrates. These studies have yielded very interesting and compelling results which indicate that an electrical signal is capable of inducing differentiation. For example, Wechsler et al.^[^ ^112^ ^]^ used indium tin oxide (ITO) glass substrates to apply a direct AC electrical signal (5–10 µA, 5–10 Hz, 1–6 h day^−1^) to hMSC, demonstrating exclusive osteodifferentiation, over chondro‐ and adipo‐differentiation, in the absence of growth factors. Substrate‐mediated stimulation of stem cells has been studied extensively with planar conductive biomaterials; electrical stimulation of thin PPy films on polystyrene (0.35 V cm^−1^, 4 h) promoted osteogenesis and mineralization of rat bone marrow‐derived mesenchymal stem cells (BMSC),^[^ ^113^ ^]^ and osteogenic differentiation was enhanced for hMSC on PANI substrates stimulated with a pulsed electrical field (7 ms, 3.6 mV cm^−1^, 10 Hz, 4 h alternating). The effect of charge injection magnitude on cellular response was investigated by Liu et al.,^[^ ^114^ ^]^ wherein a charge injection of 0.08 µC (in a range of 0.01– 0.4 µC) elicited the strongest osteogenic response of osteoblast precursor MC3T3‐E1 cells (**Figure** **9**A–C). Furthermore, this study also demonstrated the sensitivity of these cells to the timing of the electrical stimulation; cells stimulated at an early stage (2–5 days) had higher ALP activity and osteocalcin (OCN) gene expression compared to those stimulated at a later stage (5–8 days). The expression of both *β*_3_‐tubulin (neurogenic marker) and RUNX2 (early osteogenic marker) were enhanced in bone‐marrow derived hMSC when electrically stimulated in the absence of induction media (Figure 9D).^[^ ^99^ ^]^ This was an interesting study as the same electrical stimulation protocol (0.3 V, 1 ms pulse at 1 Hz) produced both a neuro‐ and osteogenic response from the cells at early time points (72 h); lineage preference was observed once spatial and topographical patterning of the graphene was incorporated, indicating a synergistic effect when combining passive and dynamic cues.

Substrate‐mediated electrical stimulation generates a directional electrical field parallel to the substrate, which has been observed to induce a migration response termed galvanotaxis. An electrical migration cue is dynamic and does not rely on functionalization of a biomaterial (i.e., chemotaxis) and the use of electric fields to dynamically guide cellular migration has been developed.^[^ ^115^ ^]^ The benefit of substrate‐mediated galvanotaxis is that is does not require the more complicated salt‐bridge or inserted electrodes approach to generate the electrical field across the cell culture. Many types of cells will migrate toward the cathode, leading to the hypothesis that the directional electrical field induces polarization of intracellular Ca^2+^ concentration.^[^ ^116^ ^]^ This does not hold true for all cell types, however,^[^ ^117^ ^]^ and so the mechanism driving galvanotaxis is not completely understood; further evidence points toward cell membrane receptor rearrangement as a possible contributing factor, in addition to polarization of lipid rafts in the cellular membrane.^[^ ^118^, ^119^ ^]^ Stem cells have demonstrated that they will undergo galvanotaxis during substrate‐mediated electrical stimulation,^[^ ^120^ ^]^ and this suggests applying this type of electrical stimulation can improve cellular penetration and population of scaffolds, without the need for chemical signals or guides.^[^ ^121^ ^]^

As the use of conductive biomaterials in tissue engineering has increased greatly, and with mounting evidence demonstrating clear, triggerable effects on proliferation, migration, and differentiation when applying a direct electrical stimulation to stem cells, more recent work has begun to focus on conductive scaffolds which can provide this stimulus on cue. As detailed in **Table** **2**, there are several electrical stimulation protocols which have been demonstrated to promote different differentiation outcomes in several multipotent stem cell sources.

**Table 2:** Conductive scaffold biomaterials delivering electrical stimulation to multipotent stem cells

Conductive biomaterial scaffold	Cells stimulated	Electrical signal	Induced response	Exogenous differentiation factors	Reference
Graphene oxide‐cellulose	ASC	Biphasic square wave, 1 V cm^−1^, 1 Hz, 200 ms, 1 h day^−1^	Proliferation, osteogenic differentiation	Yes, osteogenic media	^[^ ^98^ ^]^
PPy coated poly(trimethylene carbonate)	ASC	Biphasic square wave, 50 µm cm^−2^, 10 Hz, 4 h day^−1^	Proliferation, smooth muscle protein expression	Yes, TGF‐*β*1	^[^ ^122^ ^]^
PPy coated poly(l‐lactic acid)	BMSC	75 mV mm^−1^, 3 h day^−1^	Osteogenic differentiation	Yes, osteogenic media	^[^ ^123^ ^]^
PPy/PCL	ADMSC	200 µA, 4 h day^−1^	Migration, osteogenic differentiation	No	^[^ ^120^ ^]^
PANI/PES	iPSC	Square wave, 1 Hz for 2 ms, 50 mV cm^−1^, 1 h day^−1^	Cardiac markers	Yes, differentiation media	^[^ ^124^ ^]^
PCL/PPy	hMSC	10 mV mm^−1^ for 8 h	Osteogenic	Yes, osteogenic media	^[^ ^125^ ^]^

Electrical stimulation of multipotent stem cells, specifically MSC and adipose stem cells (ASC), primarily are reported to enhance osteogenic differentiation,^[^ ^98^, ^120^, ^123^, ^125^ ^]^ as well as smooth muscle cells as investigated by Björninen et al.^[^ ^122^ ^]^ Zhang et al. observed significant increase in osteogenic gene expression and calcium deposition of adipose derived MSCs (ADMSC) when electrically stimulated in a PCL/PPy scaffold.^[^ ^120^ ^]^ Pluripotent stem cells such as induced pluripotent stem cells (iPSC) have also demonstrated a marked response to electrical stimulation, with focus on promoting cardiac differentiation. Mohammadi et al. observed that unidirectional electrical stimulation along aligned PANI/poly(ether‐sulfone)(PES) scaffolds efficiently pushed the iPSC toward cardiomyocyte derivation.^[^ ^124^ ^]^ This focus on exploring direct electrical stimulation for osteogenic differentiation is often driven by the clinical success demonstrated in using electrical field stimulation to promote bone growth. Bone tissue is pizeoelectric in nature,^[^ ^126^ ^]^ hence it is unsurprising that an electrical signal may provide an osteogenic cue to stem cells. Myogenic, cardiogenic, and neurogenic tissue also utilize electrical signals for cell–cell communication, hence providing these signals in a controlled microenvironment may also induce differentiation toward these cell lineages. There are several theories as to how the electrical stimulation can influence differentiation; calcium signaling within cells has been demonstrated to be highly sensitive to applied electrical signals and fields, with calcium oscillation in hMSC induced or inhibited with electrical field stimulation.^[^ ^127^ ^]^ Ca^2+^ is one of the most important messengers within the cell, and is involved in many mechanisms and processes. Voltage gated ion channels in ADMSC were activated by electrical stimulation leading to higher calcium deposition over a 21 day period,^[^ ^120^ ^]^ and are another cellular component immediately affected by an electrical signal. Reactive oxygen species (ROS) generation triggered by electrical stimulation has been linked to initiating angiogenesis and cardiomyogenesis of stem cells,^[^ ^128^ ^]^ as well as osteogenesis of MC3T3‐E1 cells.^[^ ^129^ ^]^ Membrane depolarization via electrical stimulation will also have a strong effect on stem cell state, enabling enhancement of their plasticity.^[^ ^130^ ^]^ Additionally, one study to date demonstrated cytoskeletal and intracellular tension changes in hMSC after exposure to a direct current electrical field.^[^ ^131^ ^]^ However, a holistic and mechanistic understanding of how these different mechanisms affect the downstream cellular response of different stem cell types to drive differentiation is still not clearly understood. As can be seen with the various electrical stimulation protocols described in Table 2, there is little coherence in the field to determine which conductivity mechanism, waveform, duration, timing, and potential range are effective and why.

### 3.4 Electrically Induced Mechanical Stimulation

**Figure 10:**
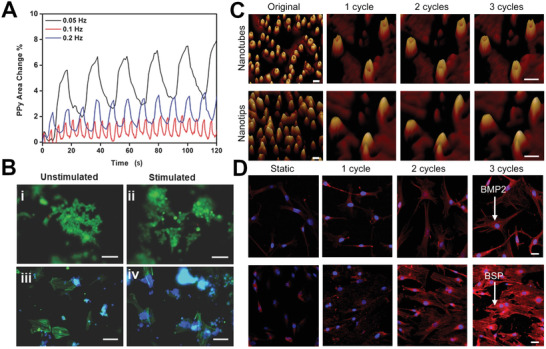
A) PPy area change percentage using optical video during applied stimulation on 30 min ECP PPy(DBS) coated fibers in PBS, with a biphasic potential waveform at 0.05 Hz (black), 0.1 Hz (blue), and 0.2 Hz (red). B‐i,ii) The unstimulated and stimulated iPSC were stained for live/dead and iii,iv) actin filaments and nuclei. Scale bars represent 100 µm. Reproduced with permission.^[^ ^84^ ^]^ Copyright 2016, Wiley‐VCH GmbH. C) Atomic force microscopy phase images in tapping mode, showing the reversible nanotube/nanotip transition due to electrochemical redox switching. Scale bars for original images, 100 nm. Scale bars for the images taken after 1–3 cycles, 20 nm. D) Immunostaining for osteogenic proteins BMP‐2 (top) and bone sialoprotein (BSP) (bottom) in MSCs cultured on nanotubes for 7 days after experiencing various numbers of cycles of the nanotube/nanotip transition. Static and 1 cycle note the weak staining of BMP2/BSP; 2 cycles and 3 cycles note the osteoblastic morphology and bright staining of BMP2/BSP. Scale bars, 20 µm. Reproduced with permission. ^[^ ^138^ ^]^ Copyright 2017, American Chemical Society.

Mechanical stimulation is an integral exogenous stimulation of the tissue engineering field; cyclic stretching and strain mimics the mechanical movement found in the cardiac environment, and hence has been demonstrated to be an effective method in targeting cardiac differentiation.^[^ ^132^ ^]^ Strain can also be used to promote alignment of cells in culture, an important aspect of osteogenesis.^[^ ^133^ ^]^ This type of stimulation is generally applied to a cell culture via bioreactors applying compression or strain. Recently, stimuli‐responsive biomaterials have been explored as an alternative to bioreactors, enabling new and flexible approaches in how to stimulate stem cells. Conductive polymers are capable of inducing mechanical stimulation to cells, transducing an electrical signal into mechanical actuation; with electrical stimulation the charge of the polymer backbone is unbalanced, and charged species will move in or out of the polymer structure, stabilizing the polymer backbone charge and generating a volume change. This mechanical response works in physiological fluids and with small potentials (range of 1 V).^[^ ^134^ ^]^ Conductive polymers, commonly PPy, have been used to directly mechanically stimulate cells in “lab on a chip” type approaches and induce increases in low piezoelectrical activity of internal calcium concentration of stimulated cells.^[^ ^135^, ^136^ ^]^ This approach of using the conductive polymer's unique actuation properties in 3D scaffolds has recently been explored; PPy coated poly(lactic‐co‐glycolic acid) (PLGA) fibers underwent cyclical swelling and shrinking when an electrical signal was applied to the scaffold (biphasic waveform 0.2 to −1 V, 0.05 Hz for 600 s every 24 h for 3 consecutive days) which delivered omnidirectional strain (6–8%) and flow through the scaffold pores to the seeded iPSC, in addition to the electrical stimulation (**Figure** **10**A,B). This dual mechanical and electrical stimulation was observed to significantly downregulate Oct4, indicating differentiation of the iPSC, and upregulation of cardiomyocyte specific genes NKX2.5 and GATA4 both with and without stimulation on the PPy/PLGA scaffolds.^[^ ^84^ ^]^ The combination of electrical field and mechanical stimulation has been applied in bioreactor set ups using acellular scaffolds, where cardiomyocyte differentiation was significantly more efficient compared to only mechanical stimulation, indicating a benefit in a synergistic stimulation approach for cardiac tissue engineering.^[^ ^137^ ^]^

A slightly different approach used conductive polymers nanostructured materials; PPy nanotubes were electrically stimulated to reversibly cycle from open to a closed morphology in order to provide mechanical stimulus to MSC (Figure 10C,D). This induced osteogenic differentiation of the MSC, with clear mechanotransduction genes triggered and osteogenic biomarkers observed when undergoing 3–5 cycles of actuation (40 min per cycle).^[^ ^138^ ^]^ Furthermore, this mechanical stimulus was only provided after 1 day of culture, after which the cells were cultured for a further 7 days with no osteogenic supplement for differentiation assessment. Hence it is clear that even a short duration of mechanical stimulation can have a significant downstream effect on stem cell differentiation.

There has also been some progress in the field of using ionic hydrogel biomaterials which undergo actuation in the presence of an electrical field; here the actuation is driven by rearrangement of ions within the hydrogel polymer structure when a field is applied. This type of stimuli‐responsive biomaterial has typically been developed for the field of biomedical soft robotics, however, they have also demonstrated biocompatible support of cells^[^ ^139^ ^]^ making them an interesting biomaterial class for skeletal muscle tissue engineering.

### 3.5 Electrically Induced Drug Release

**Figure 11:**
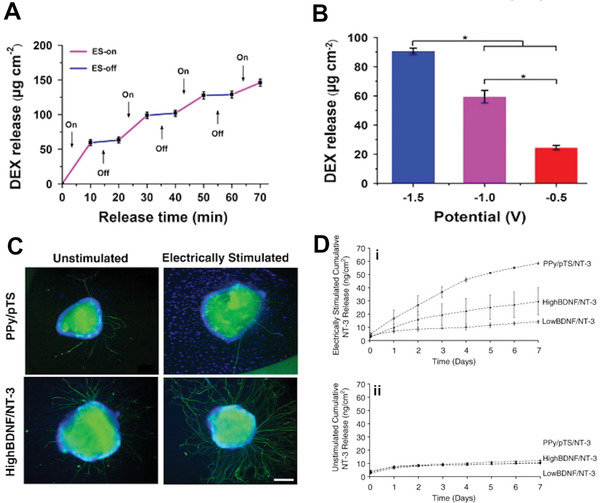
A) On‐demand delivery profile of dexamethasone from PDA‐PPy‐MCs under −1 V. B) Amount of dexamethasone released from the PDA‐PPy‐MCs under different potentials for 10 min. Reproduced with permission.^[^ ^149^ ^]^ Copyright 2017, Springer Nature. C) Representative images of cochlear neural explants grown on PPy/pTS polymers with and without neurotrophin. Neurites were visualized by immunocytochemistry with a neurofilament‐200 primary antibody and a fluorescent secondary antibody (green). Cell nuclei are labelled with DAPI (blue). Scale bar is 200 µm and the same for all images. D) Release of NT‐3 from PPy/pTS films: a) with electrical stimulation, and b) without electrical stimulation. The release of NT‐3 from a PPy film containing only NT‐3 neurotrophin (PPy/pTS/NT‐3) is compared to the release of NT‐3 from PPy/dual neurotrophin films. Dual neurotrophin films were grown with both neurotrophins at 2 µg mL^−1^ (“HighBDNF/NT‐3”) or with both neurotrophins at 1 µg mL^−1^ (“LowBDNF/NT‐3”), while single films were grown with NT‐3 at 2 µg mL^−1^. Each point represents the average of three measurements with error bars representing the standard deviation. Reproduced with permission. ^[^ ^152^ ^]^ Copyright 2010, Elsevier.

Electrically responsive biomaterials are capable of drug release, with several mechanisms triggered by an electrical signal enabling localized responsive and controlled drug release.^[^ ^140^, ^141^ ^]^ Conductive polymers have been widely studied for controlled drug release, as anionic molecules (dopants) can be easily loaded into the polymers during electrochemical synthesis and then released as a reducing electrical potential is applied.^[^ ^142^ ^]^ As the choice of dopant molecule is limited by charge and weight, this enables a two‐step process in functionalizing the polymer with a wider range of molecules for controlled delivery. Cationic molecules can also be loaded into the polymer through a three‐step loading process once the polymer has been synthesized. There is also typically some passive diffusion from the materials when in an electrolytic solution due to ion exchange. The drug release via electrical stimulation is also generally quite rapid, and so while this is useful for localized burst release, this is less beneficial for long term implants. Many different drugs and growth factors have been incorporated into conductive polymers for controlled release, including dopamine,^[^ ^143^ ^]^ naproxen,^[^ ^144^ ^]^ heparin,^[^ ^145^, ^146^ ^]^ nerve growth factor (NGF),^[^ ^147^ ^]^ and dexamethasone.^[^ ^148^, ^149^, ^150^ ^]^ Biotin has also been used as a dopant in conductive polymers, creating surface receptors for functionalization via streptavidin which can then be released upon electrical stimulation.^[^ ^151^ ^]^ Using electrical stimulation, the drug release can be controlled step‐wise, as well as control of the payload volume through modification of the potential used (**Figure** **11**A,B). Controlled drug release for stem cell control has not been widely explored using this system, with most of the focus on NGF and brain‐derived neurotrophic factor (BNDF) release for neural stem cells and nerve cells. The synergistic combination of electrical stimulation and drug release has demonstrated significant effect on neuronal cell development and growth (Figure 11C,D).^[^ ^152^, ^153^ ^]^ The drug loading capacity of these electrically responsive biomaterials can also be increased through creating porous and high surface area scaffolds,^[^ ^154^, ^155^ ^]^ or through creating composite materials. A gelatin‐oligoaniline nanofibrous scaffold demonstrated controlled dexamethasone release when electrically stimulated, as well as supporting MSC growth and proliferation.^[^ ^156^ ^]^ The actuative ability of conductive polymers (detailed in Section 5.1) can also be used as an electroactive switch for releasing drugs loaded with nanotube structures, opening the nanotubes for drug release upon electrical stimulation.^[^ ^157^ ^]^ An electroactive scaffold demonstrated controlled release of human BMP‐4 in a rabbit animal model, where electrodes were inserted across a bone defect to induce the controlled release. Rapid, high quality bone healing was observed with a synergistic benefit of both the drug release and the electrical stimulation.^[^ ^158^ ^]^ Further exploration into incorporating osteogenic or cardiogenesis growth factors in combination with the electrical stimulation described in the previous section may also benefit from this synergistic approach. Even the incorporation of an anti‐inflammatory drug such as dexamethasone may be beneficial as a burst drug release upon implantation in vivo, and help to control the initial microenvironment for stem cells once implanted.

Electrically responsive hydrogels are used for drug release, wherein an electric field can be applied to release the drug from the hydrogel.^[^ ^159^ ^]^ The main electrical stimulation mechanism this is achieved through is via deswelling of the hydrogel due to rearrangement of charged molecules, thus ejecting the drug from the polymer network. This effect was demonstrated with the controlled, pulsatile release of dexamethasone from an electroactive hydrogel.^[^ ^160^ ^]^ These hydrogels were also seeded with PC12 cells with good adhesion and proliferation, further illustrating the potential for dexamethasone loaded electrically responsive scaffolds in tissue engineering.

### 3.6 Electrically Controlled Surface Presentation

Electrical stimulation can be used to control the presentation of surface molecules on electrically responsive biomaterials. As conductive polymers can be oxidized or reduced upon electrical stimulation, this will change the surface energy of the polymer and modify how charged molecules interact with the surface, and thusly how cells will adhere to the biomaterial.^[^ ^161^, ^162^, ^163^ ^]^ This can be used to “precondition” the conductive polymer surface to electrostatically bind ECM proteins in a beneficial conformation prior to cell seeding^[^ ^164^, ^165^ ^]^ and has been used to influence cellular behavior in a pretreated scaffold.^[^ ^166^ ^]^ This redox switching has also been used to some extent to exert temporal control over adherent cells; reducing the conductive polymer surface during cell culture resulted in the detachment and reduced viability of NSC.^[^ ^167^ ^]^

Changing the redox state of the conductive polymer can also expose a higher density of dopant at the surface; electrical stimulation of PPy doped with heparin exposed up to three times more heparin on the polymer surface.^[^ ^146^ ^]^ The electrical stimulation used here reduced the polymer backbone, hence the negatively charged sulfate groups within the heparin structure were available for interaction and no heparin was released from the polymer structure. While there is not evidence that this electroactive switching can be used for temporal control once cells are adherent, there are possibilities in using electrical stimulation to control the surface presentation of ECM proteins for initial control of cellular adhesion. A “pixel” or patterning approach using the redox state of a polymer was proposed as a pathway for control of cellular adhesion,^[^ ^164^ ^]^ and could be an interesting approach in spatial control on biomaterial scaffolds.

### 3.7 Piezoelectric Mediated Electrical Stimulation

Biocompatible piezoelectric materials are increasingly being developed for applications in biomedicine and tissue engineering due to their interesting properties; typically, the piezoelectric effect is used to deliver electrical stimulation upon wireless mechanical stimulation. Piezoelectric materials can generate a strain when an electrical input is applied, and conversely, can generate an electrical output when strain is applied; this occurs due to a net dipole moment when mechanical stress induces a transient deformation of the material crystal structure. This ability to induce an electrical signal through a noninvasive, external trigger makes piezoelectric scaffolds an intriguing approach to new smart biomaterial scaffolds for tissue engineering.^[^ ^168^, ^169^ ^]^

Biocompatible piezoelectric materials encompass a range of synthetic polymers such as poly(vinylidene fluoride) (PVDF), the copolymer vinylidene fluoride‐trifluoroethylene (PVDF‐TrFE), poly‐3‐hydroxybutyrate‐3‐hydroxy valerate (PHBV), natural polymers such as collagen, cellulose, and chitin, and biocompatible ceramics such as barium titanate (BT), zinc oxide, and boron nitride.^[^ ^169^, ^170^ ^]^ Of these, PVDF and PVDF‐TrFE are commonly used as these polymers are ideal for biomaterial applications as they are flexible and are easily processable. Similar to conductive polymers, they can be combined with other polymer materials, electrospun into fibers or processed into porous scaffolds. These polymers must also be “poled” in order to facilitate pizeoelectric activity, hence “unpoled” polymers are a simple but effective control to study the pizeoelectric effect on cells.

Wireless mechanical stimulation of piezoelectric materials can be achieved using ultrasound, thus enabling controlled generation of an electrical signal.^[^ ^171^ ^]^ 3D piezoelectric scaffolds enhanced the osteogenic differentiation of SaOS‐2 bone‐like cells, with increased COL1 expression and deposition of hydroxyapatite on ultrasound stimulated scaffolds.^[^ ^172^ ^]^ Human umbilical cord MSCs demonstrated enhanced proliferation and ALP activity when cultured on PLLA/graphene/BT scaffolds and stimulated with external ultrasound.^[^ ^173^ ^]^ Poled *β*‐PVDF films mechanically stimulated via vertical vibration (1 Hz, ≈1 mm height) demonstrated enhanced osteogenic differentiation of hASC; ALP activity on the poled PVDF films with mechanical stimulation and without osteogenic media was significantly greater than the static samples, and mechanical stimulation with osteogenic media showed a further increase.^[^ ^174^ ^]^ The pizeoelectric activity of a material was found to be an important factor when studying MSC differentiation; 3D PVDF‐TrFE fibrous scaffolds with higher levels of piezoelectric activity promoted osteogenesis, while lower levels promoted chondrogenesis when undergoing dynamic loading.^[^ ^175^ ^]^ This is an interesting effect, demonstrating that tuning the piezoelectric properties can be used to preferentially target different lineages from the same stem cell source.

## 4 Ultrasound Activation

Ultrasound, pressure waves oscillating at frequencies at or above 20 kHz, can be employed as a powerful remote stimulus for biomaterials. The oscillating pressure^[^ ^176^, ^177^ ^]^ and resulting mechanical effects generated by ultrasound have been harnessed for various biomedical applications including controlled release from acoustically responsive carriers,^[^ ^14^, ^178^, ^179^, ^180^ ^]^ acoustically triggered hydrogelation,^[^ ^181^ ^]^ enhancement of agent transdermal permeability via sonophoresis,^[^ ^182^ ^]^ in vitro manipulation of cells into defined geometric assemblies,^[^ ^183^ ^]^ and the temporary disruption of biological barriers to facilitate drug entry.^[^ ^184^, ^185^ ^]^ Generated mechanical effects of relevance to delivery include acoustic cavitation, which is the forced size oscillation or growth and collapse of gas microbubbles within a fluid,^[^ ^186^ ^]^ as well as related fluid streaming.^[^ ^187^, ^188^, ^189^, ^190^ ^]^ Ultrasound waves penetrate readily through biological tissue and tissue‐like constructs and can be applied in a focused manner,^[^ ^191^, ^192^ ^]^ allowing for in situ manipulation of materials and cells deep within. By controlling the intensity and duration of the applied energy, ultrasound can be employed with high biocompatibility.^[^ ^192^ ^]^ While low intensity pulsed ultrasound itself has previously been studied as a means of influencing osteogenic differentiation,^[^ ^193^, ^194^ ^]^ here we highlight examples of controlled ultrasound manipulation of engineered biomaterials, primarily for growth factor delivery. The design of scaffolds capable of in situ cellular control via ultrasonic stimuli is an emerging area of materials development.

Regulating the temporal presentation of growth factors and other bioactive molecules is key for controlling cellular response^[^ ^195^ ^]^ and, ultimately, the regenerative capacity of an engineered tissue therapy. To this end, there has been interest in developing hydrogel scaffolds which incorporate ultrasound‐controlled payload release systems for on‐demand controlled delivery schedules; several of these material systems are summarized in **Table** **3**. These scaffolds can be designed to liberate growth factors or bioactive payloads from a hydrogel matrix upon acoustic stimulation.

**Table 3:** Ultrasound‐responsive scaffold systems for controlled release of bioactive factors

Material	Ultrasound application	Material response	Cell type	Cell response	Reference
Alginate‐wall capsules containing AuNP or iron oxide microparticle payload, integrated into larger bulk alginate hydrogel	20 kHz probe sonicator, amplitude and duration varied	Release of particle payload from capsules; US‐tunable release for “weak” versus “strong” capsules	D1 mMSCs	Increased osteogenic (ALP) activity (upon addition of AuNP‐BMP to plated cells)	^[^ ^198^ ^]^
Alginate hydrogel microbeads with sterically entrapped AuNP‐BMP	20 kHz probe sonicator, 9.6 mW cm^−2^, 2.5 min h^−1^ for 10 h (for AuNP‐BMP)	Release of AuNP‐BMP from hydrogel‐microbeads	D1 mMSCs	Increased osteogenic (ALP) activity (from supernatant of stimulated material)	^[^ ^199^ ^]^
Fibrin hydrogels containing double emulsion particles (water‐in‐PFC‐in‐water) loaded with bFGF	3.5 MHz, 10 cycles, 10 ms pulse repetition rate with 12.9 MPa peak compressional pressure, 6.0 MPa peak rarefactional pressure	Release of bFGF from double emulsion particles	HUVECs^a)^; mouse C3H10T1/2 mesenchymal progenitor line	bFGF releasates from the ultrasound exposed hydrogels increased HUVEC metabolic activity; no viability reduction with ADV (both cell types)	^[^ ^200^ ^]^
Fibrin hydrogels containing double emulsion particles (water‐in‐PFC‐in‐water) loaded with bFGF	2.5 MHz,13 cycles, 100 Hz pulse repetition frequency, 4–8 MPa peak rarefactional pressure	Release of bFGF from double emulsion particles	Endogenous cells around subcutaneous implant (mouse)^a)^	Increased vascularization in and around implanted scaffolds	^[^ ^201^ ^]^
Calcium‐crosslinked alginate hydrogels with noncovalently complexed agent	20 kHz probe sonicator; 120 mW cm^−2^, 2.5 min day^−1^ (for in vivo study); in vitro studies at 9.6 mW cm^−2^; interval between applications varied	Release from gel of mitoxantrone (for in vivo work), VEGF‐165, naproxen, SDF‐1*α*, and plasmid DNA; pulsed release	MDA‐MB‐231 breast cancer mouse tumor xenografts^a)^	Tumor size reduction with daily short, high‐dose mitoxantrone	^[^ ^206^ ^]^
Calcium‐crosslinked alginate hydrogels with loaded agent	20 kHz probe sonicator, amplitude and duration varied; repeated pulses	Release from gel of VEGF, PDGF, mitoxantrone; pulsed release; sequential release of 5FU and irinotecan	B16F10 mouse melanoma cells^a)^	5FU followed by irinotecan exposure reduced cell populations upon addition of free drug in plated culture	^[^ ^207^ ^]^

^a)^ Non‐stem cells.

### 4.1 Ultrasound‐Responsive Carriers for Scaffold Incorporation

One strategy employed to generate ultrasound‐responsive scaffold materials involves the incorporation of acoustically responsive delivery particulates within a gel matrix. Delayed delivery of various growth factors may have a beneficial effect in tissue engineering applications including bone regeneration.^[^ ^196^, ^197^ ^]^ Thus, requirements of a suitable material include high payload retention for an extended duration coupled with the ability to respond to an ultrasound trigger to release payload at the desired time and rate.

**Figure 12:**
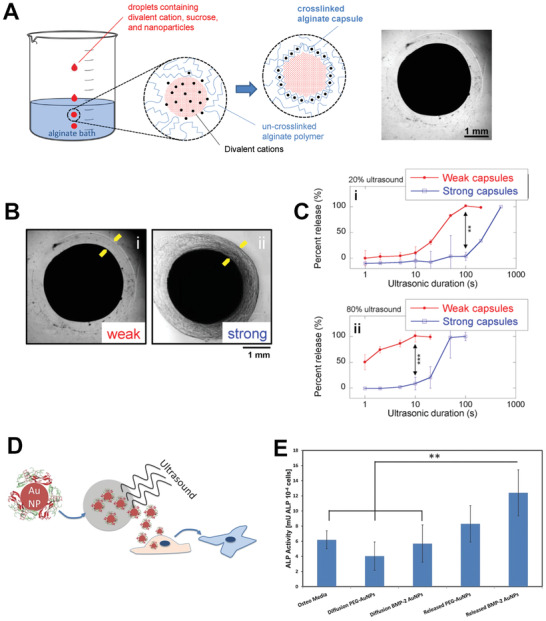
Ultrasound‐responsive carriers. A) Tunable ultrasound‐rupturable, alginate‐walled capsules are formed when droplets with divalent cations, sucrose and payload nanoparticles are submerged in an alginate bath, causing the divalent cations to interact with the alginate and form a crosslinked capsule wall around the payload. Microscopy image of an iron oxide particle‐loaded capsule is shown at right. B) Microscope images of i) “weak” capsules formed from 50 × 10^−3^ m CaCl_2_ and ii) “strong” capsules formed from 100 × 10^−3^ m BaCl_2_ with a thicker wall. C) Percent of gold nanoparticle (AuNP) payload release from weak and strong capsules as a function of ultrasound exposure time at i) 20% and ii) 80% ultrasound amplitudes (mean +/− standard deviation), ** *p* < 0.01, *** *p* < 0.001. Adapted with permission.^[^ ^198^ ^]^ Copyright 2015, Elsevier. D) Schematic of approach using alginate microbeads with sterically entrapped payload: ultrasound stimulates release of AuNP functionalized with BMP‐2 from alginate microbeads and the release medium is added to D1 cell cultures. E) D1 cell ALP activity measured at day 7 for cells treated with diffusion‐released and ultrasound‐released (9.6 mW cm^−2^, 2.5 min per h for 10 h) AuNP payload from alginate microbeads, showing bioactivity of the released BMP‐2 AuNPs (mean +/− standard deviation), ^**^ *p* < 0.015. Adapted with permission.^[^ ^199^ ^]^ Copyright 2015, Wiley‐VCH GmbH.

To address these requirements, Kennedy et al. developed high‐retention, ultrasound‐burstable capsules which can be integrated into bulk hydrogels for triggered release.^[^ ^198^ ^]^ Alginate‐walled 4 mm diameter capsules were formed to encapsulate nanoparticle payload solutions (**Figure** **12**A). These capsules showed near‐complete retention of gold nanoparticle (AuNP) payload over 7 days at which point ultrasound exposure resulted in nearly 100% payload release. By using different divalent cations to crosslink the alginate (either calcium or barium chloride), the authors were able to modulate the mechanical properties and thickness of the capsule walls, creating “strong” versus “weak” capsules (Figure 12B). BaCl_2_ resulted in stronger capsule walls that required higher ultrasound exposure (i.e., longer duration at a given intensity) to rupture (Figure 12C). By mixing these two particle types, the authors demonstrated the ability to rupture the weak capsules with lower intensity ultrasound with minimal release from the stronger‐walled capsules. The stronger capsules were subsequently ruptured with higher intensity ultrasound. The alginate‐wall capsules were also demonstrated to rupture when integrated into larger bulk alginate gels and placed within chicken tissue. To create a bioactive payload, AuNP were functionalized with BMP‐2 (AuNP‐BMP), which induced osteogenic differentiation of mouse bone marrow stromal mesenchymal stem cells (D1 mMSCs). D1 mMSCs within alginate gels did not show significant viability reduction upon ultrasound exposure, supporting the biocompatibility of this technique. For application to bone regeneration, the ability of these capsules to induce differentiation upon ultrasound stimulation is an exciting area of future exploration.

Steric hindrance is another design strategy which can be used to achieve high retention of bioactive payloads within carriers prior to ultrasound‐induced release. Using this strategy, Kearney et al. demonstrated high retention rates of pegylated AuNP physically entrapped within ionically crosslinked alginate microbeads (250 µm diameter) prior to ultrasonic stimulation.^[^ ^199^ ^]^ The pore size of the alginate hydrogels allowed for steric entrapment of 30–100 nm diameter nanoparticles within the microbeads. Ultrasound exposure disrupted the microarchitecture of the hydrogel microbeads, freeing entrapped nanoparticles without detectable gel fragmentation. A 200‐fold increase in release rate was demonstrated for samples ultrasound‐stimulated at 24 h postencapsulation compared to diffusion alone. This steric hindrance strategy did not rely on charge interactions between the alginate and nanoparticles for retention. To investigate bioactive nanoparticle payloads, AuNP functionalized with BMP‐2 were entrapped in the microbeads and exposed to ultrasound, with the resulting supernatant administered to D1 mMSCs (Figure 12D). Bioactivity of the ultrasound‐released AuNP‐BMP payload was demonstrated via increased ALP activity of D1 mMSCs compared to osteogenic media (Figure 12E). These microbeads show promise for ultrasound‐induced delivery of nanopayloads to stem cells either as particulates dispersed within a bulk scaffold or directly injected in vivo for ultrasound‐stimulated control.

Acoustically responsive emulsions are another class of carriers that have been incorporated within scaffolds for controlled payload release. Fabiilli et al. demonstrated the successful incorporation into fibrin hydrogels of perfluorocarbon (PFC) double emulsion particles loaded with basic fibroblast growth factor (bFGF).^[^ ^200^ ^]^ The double emulsion comprised a water‐in‐PFC‐in‐water structure (W_1_/PFC/W_2_) where the bFGF was loaded into the W_1_ phase and the hydrophobic PFC prevented its release from the particle into the surrounding W_2_ phase. Exposure to ultrasound caused acoustic droplet vaporization (ADV) of the PFC creating gas bubbles that disrupted the emulsion structure, releasing the bFGF from the W_1_ phase. This resulted in a fivefold increase in bFGF release. Human umbilical vein endothelial cells (HUVECs) showed increased metabolic activity when exposed to bFGF released from the gels thus demonstrating the bioactivity of the released proteins. Importantly, ultrasound‐induced ADV did not significantly reduce the viability of HUVECs or a mouse C3H10T1/2 mesenchymal progenitor line cultured within these droplet‐hydrogel scaffolds. The reaction of the emulsions to the ultrasound and the resulting bubble formation also altered the structural properties of the fibrin hydrogel. Gels exposed to ultrasound increased their shear stiffness 22‐fold due to the consolidation of the fibrin and bubble formation. This scaffold design could potentially be leveraged for stem cell control in the future via controlled release of growth factors and/or temporal manipulation of scaffold mechanical properties.

Moncion et al. demonstrated the use of a microfluidic flow‐focusing system to obtain a high level of control over the size distribution of double emulsion particles.^[^ ^201^ ^]^ Particles with the W_1_/PFC/W_2_ geometry were synthesized by first forming the W_1_/PFC emulsion with bFGF payload in the W_1_ phase, and then injecting this into the central channel of a flow‐focusing chip to create the double emulsion. These ultrasound activatable particles were incorporated into a fibrin hydrogel mixture and injected subcutaneously in mice and allowed to polymerize. The emulsion‐loaded hydrogel was then exposed to ultrasound for 2 min on a daily basis for 7 or 14 days starting one day after implantation. Ultrasound‐stimulation induced rupture of the emulsions through bubble formation, releasing bFGF protein into the gel where it diffused out into the surrounding tissue and was able to stimulate blood vessel growth. The level of perfusion in the region around the ultrasound‐stimulated gels was significantly higher than regions around unstimulated control gels. Gels that received ultrasound also had the highest density of blood vessels, supporting the use of this platform for in vivo cell manipulation using triggered growth factor release.

### 4.2 Ultrasound‐Induced Manipulation of Hydrogel Scaffold Crosslinks

Ultrasound stimulation can also be used to manipulate crosslinks within a hydrogel material to influence the temporal profile of payload release. The ability to specify the release rate of bioactive cue payloads is a desirable feature for stem cell control.^[^ ^202^ ^]^ Such a system could enable an improved understanding of the complex, transient presentation of cues during tissue regeneration or developmental processes^[^ ^203^, ^204^ ^]^ and enable recapitulation of these cues within implanted engineered tissues. While release rate can be controlled to some extent via degradable hydrogels or passive outward payload diffusion,^[^ ^205^ ^]^ these strategies preclude on‐demand temporal control.

**Figure 13:**
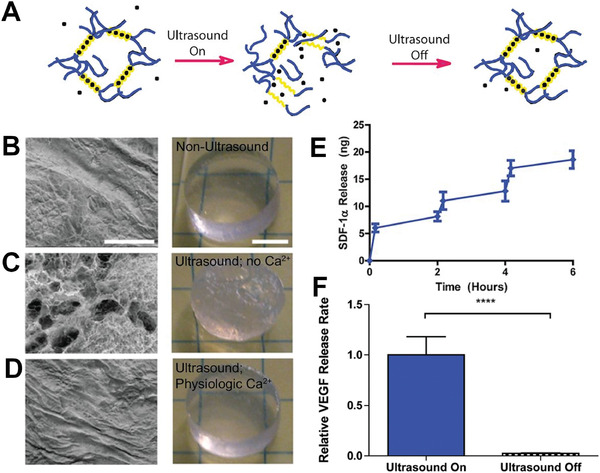
Ultrasound‐induced manipulation of hydrogel crosslinks. A) Schematic of the proposed mechanism of ultrasound‐induced transient disruption and self‐healing of calcium‐crosslinked alginate hydrogels. After gel disruption, calcium (black dots) within physiologic fluids can reform crosslinks via binding to alginate polymer guluronic acid chains (yellow). B) Scanning electron microscopy (SEM) images (left) and gross structure photographs (right) of calcium alginate hydrogels before ultrasound, C) after ultrasound treatment (9.6 mW cm^−2^, 5 min pulses, once per hour) without divalent cations in the medium, and D) after ultrasound treatment in medium with physiologic levels of calcium ions. Scale bars (B)–(D): 200 µm in SEM images; 5 mm in photographs. E) Cumulative release of SDF‐1*α* from gels exposed to ultrasound (9.6 mW cm^−2^) for 10 min every 2 h. F) VEGF‐165 (^125^I‐labeled) relative release rate from alginate gels with and without ultrasound treatment (mean +/− standard deviation), ^****^ *p* < 0.001. Adapted with permission.^[^ ^206^ ^]^ Copyright 2014, National Academy of Sciences.

Ultrasound is well‐suited as a stimulating trigger for implantable scaffolds due to its significant tissue penetration. Many ultrasound‐responsive systems result in a permanent change to the delivery vehicle with a single ultrasound exposure, thus hindering the generation of pulsatile release profiles with sequential applications of the ultrasound trigger. Huebsch et al. address this challenge by designing ionically crosslinked alginate hydrogels where ultrasound exposure can transiently increase the rate of drug release, allowing for sequential triggered‐release periods.^[^ ^206^ ^]^ Ultrasound stimulation disrupts the crosslinking of the guluronic acid chains of the alginate polymers, thus enhancing payload release (**Figure** **13**A). Upon ultrasound termination, the crosslinks are reformed through binding of calcium present in the surrounding physiological fluids thereby reducing the release rate. This re‐linking prevents permanent damage to the gel (Figure 13B–D). Additional ultrasound exposures repeated this cycle thereby enabling nearly “digital” on/off release of payload to create temporally short release periods of high concentration drug. The authors showed pulsatile release of extracellular matrix‐binding cytokine stromal cell derived factor 1*α* (SDF‐1*α*) as well as on‐demand release of vascular endothelial growth factor 165 (VEGF‐165) (Figure 13E,F). The authors also explored chemotherapy applications of this system, showing that this pulsatile release pattern enhanced the toxicity of the chemotherapy agent mitoxantrone toward MDA‐MB‐231 and MCF7 breast cancer cells. Demonstrating in vivo feasibility, they further showed in a xenograft mouse tumor model that a daily burst release of mitoxantrone triggered by ultrasound exposure to implanted hydrogels near the tumor showed greater reduction in tumor growth compared to continuous release alone. Though the in vivo work was applied to tumor therapy, the ability to control release profiles over extended multiday time periods could also prove a powerful tool for influencing and studying stem cell response to temporal growth factor patterns.

Another challenge that hydrogel‐based drug delivery systems face is controlling the release rate of different compounds from the same gel. Addressing this challenge, Emi and coauthors utilized a similar self‐healing calcium‐crosslinked alginate hydrogel system that allowed pulsatile payload release in response to ultrasound.^[^ ^207^ ^]^ Individual payloads showing enhanced ultrasound‐stimulated release included the signaling proteins VEGF and platelet derived growth factor (PDGF) as well as the chemotherapy agents irinotecan, mitoxantrone, and 5‐fluorouracil (5FU). Single ultrasound pulse exposures that resulted in drug release from the gels were often long enough in duration to also result in heating and gel erosion. Pulsing the ultrasound exposure reduced these effects while still allowing payload release from the gel. The authors also demonstrated sequential release of multiple payloads (irinotecan and 5FU) from the same hydrogel construct. Here they designed the gel to release 5FU initially through passive outward diffusion alone but retain most of the irinotecan. At 18 h ultrasound was used to enhance the release rate of the irinotecan. Different delivery schedules of both drugs were shown to result in distinct effects on the survival of B16F10 mouse melanoma cancer cells. Utilizing this pulsed ultrasound stimulation platform for control of multiple growth factor payloads could provide a useful tool for stem cell manipulation by creating distinct temporal exposure profiles of individual cues.

Scaffolds for ultrasound‐induced growth factor release hold exciting potential to allow the on‐demand remote‐controlled delivery of bioactive cues from a tissue‐engineered construct and to enable controlled‐release in vivo postimplantation. Careful characterization of the applied ultrasound dosage and investigation of extended material biocompatibility and stability will be key for in vivo implementation of ultrasound‐controlled materials for stem cell manipulation.

## 5 Magnetic Field

Magnetically responsive composite materials are an interesting category of stimuli‐responsive biomaterials; an externally applied magnetic field can induce targeted and controlled stimuli with spatial accuracy, and with excellent tissue penetration. Hence magnetically responsive biomaterials are a promising class of stimuli‐responsive scaffolds for in vivo application, enabling noninvasive controlled stimulation after implantation. Primarily static magnetic fields (SMF) and pulsed electromagnetic fields (PEMF) are used when applying a magnetic field to cells, tissue, and magnetically responsive biomaterials. Externally applied magnetic fields have been used, without the presence of magnetically responsive materials, to promote cellular proliferation and differentiation with a particular focus on bone tissue formation and repair.^[^ ^208^ ^]^ SMFs have been shown to influence differentiation and proliferation of hADMSC^[^ ^209^ ^]^ and dental pulp stem cells.^[^ ^210^ ^]^ PEMFs have been demonstrated to have an influence on cell proliferation and differentiation.^[^ ^211^, ^212^, ^213^, ^214^ ^]^ Hence the presence of the magnetic field, coupled with the magnetically responsive scaffolds, may promote a synergistic therapeutic response in stem cells for tissue repair.

Magnetically responsive biomaterials are typically biocompatible materials coupled with magnetic nanoparticles (MNPs), either loaded into or coated onto the material. MNPs in biomedical applications are generally superparamagnetic magnetic nanoparticles, and magnetite (Fe_3_O_4_) and maghemite (*γ*‐Fe_2_O_3_) are commonly used due to their biocompatibility.^[^ ^215^ ^]^ A biocompatible composite magnetic scaffold was first developed by Bock et al. wherein a scaffold of hydroxyapatite and collagen was dip‐coated with magnetite MNP.^[^ ^216^ ^]^ This scaffold supported MSC derived from hBMSC, demonstrating biocompatibility and capability for supporting osteogenic differentiation. Since then, a variety of magnetically responsive biocompatible scaffolds have been developed to explore using magnetic fields to stimulate cells. Natural and synthetic polymers have been used, and with careful consideration of the MNP properties, scaffold processing such as electrospinning^[^ ^217^ ^]^ and 3D printing^[^ ^218^ ^]^ can be facilitated hence quite a range of designs can be achieved.

There are two main pathways in which magnetically responsive biomaterials can deliver stimulation to cells upon the application of a magnetic field; mechanical actuation/stress induced by local MNPs, and release of growth factors/bioactive molecules. In these approaches, the mechanical stimulus can be applied as required as the stimulation is facilitated by actuation of the MNPs within the scaffold, and the molecular release is controlled and localized but finite.

### 5.1 Magnetically Induced Mechanical Stimulation

Mechanical stimulation via MNPs is induced when a magnetic field is applied and the MNPs move to align their dipoles with the field. Intracellular MNP have been used to deliver local mechanical stress, directly stimulating cells to promote osteoblast differentiation.^[^ ^219^, ^220^ ^]^ The ability of MNPs in a magnetic field to induce physical changes within a scaffold was demonstrated by Hao et al. where nanodeformation of the PLGA/MNP scaffold was observed using AFM and the expression of Pizeo1 (mechanosensitive gene) was increased in the magnetically stimulated cells. While the reported nanodeformation was small (feature height changes of 40–60 nm), nanoscale actuation has been demonstrated to have a significant influence on cellular behavior through mechanotransduction pathways.^[^ ^221^ ^]^ This approach has been used in several magnetically responsive scaffolds, detailed in **Table** **4**, to stimulate cells in order to promote primarily an osteogenic response. In each of these studies, the magnetic field stimulation combined with a magnetically responsive scaffold induced the strongest response from the cells.

**Table 4:** Magnetically responsive scaffolds stimulating multipotent and primary cells

Material/MNP	Magnetic field	Cell type	Response to stimuli	Reference
PLGA/IO‐OA	70–80 mT SMF	MC3T3‐E1	Increased calcium deposition and ALP/OCN/BMP2 expression	^[^ ^222^ ^]^
PEG/MNP	50 mT SMF	SVF	Increased ALP/RUNX2/COL1/OST activity, and increased VEGF levels	^[^ ^223^ ^]^
Chitosan/Fe_3_O_4_	2.69–2.95 mT, inductive coupling force	7F2 osteoblasts	Increased proliferation and COL1/ALP	^[^ ^218^ ^]^
PCL/Fe_3_O_4_	25–30 mT, 70 Hz sinusoidal	hMSC	Increased ALP activity	^[^ ^224^ ^]^
PCL/Fe_3_O_4_	15 mT SMF	Primary mouse osteoblasts	Increased ALP/RUNX2/OST expression	^[^ ^225^ ^]^
PCL/DT‐NP	0.30 T SMF	hASC	Enhanced tenogenesis	^[^ ^226^ ^]^

**Figure 14:**
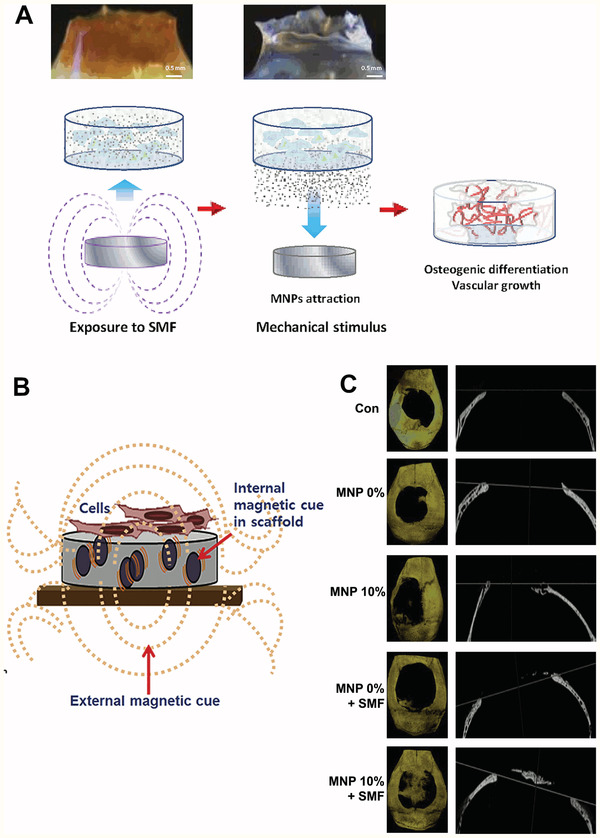
A) Schematic diagram of nanoparticle release from gels under the magnetic gradient. Reproduced from^[^ ^223^ ^]^ Copyright 2019, Elsevier. B) Schematic illustration showing the combined magnetic cues from internal magnetic nanoparticles and the external magnetic field that can influence the cell responses. C) Representative micro CT images constructed for the calcified tissues in the defects (top view; left, and lateral cross‐section view; right). Reproduced with permission.^[^ ^225^ ^]^ Copyright 2016, Elsevier.

Tomás et al. created yarn scaffolds of polycaprolactone (PCL)/DT‐NP and seeded them with hASC, and when an SMF was applied, a significant increase in the expression of tendon‐related markers was observed. The mechanical actuation of the magnetically responsive yarns was attributed to stimulating the stem cells and boost their tenogenetic differentiation. This use of magnetically responsive scaffolds has seen an increase in interest for tendon tissue engineering, due to the mechanotransduction cues the scaffolds can provide.^[^ ^227^ ^]^ Filippi et al. developed a PEG/MNP hydrogel system did not functionalize the MNPs to the scaffold polymer, instead allowing the MNP to migrate through the hydrogel. Magnetic stimulation was used to precondition a heterogenous population of stromal vascular fraction (SVF) cells prior to implantation, inducing both osteogenic and vasculogenic properties of the cells. The migration of the MNP through the hydrogel material facilitated the formation of dense osteoblastic tissue in vitro, similar to in vivo tissue. Additionally, endothelial cells were observed to form vascular structures in the soft hydrogel after undergoing magnetic stimulation, an important step in developing engineered tissue. This was an interesting approach to develop a coculture of targeted cell lineages with a relatively simple cue delivered by the magnetic field stimulation. Furthermore, an SMF was applied to migrate the MNPs out of the hydrogel structure prior to in vivo implantation into a mouse model (**Figure** **14**A).^[^ ^223^ ^]^ As the toxicity of MNP may be problematic, this is an interesting approach to using magnetic stimulation with minimal long term risks postimplantation. Furthermore, with the relative ease of applying an external magnetic field and excellent tissue penetration,^[^ ^223^ ^]^ these responsive scaffolds have already demonstrated success in vivo. Osteogenic differentiation of mouse calvarial osteoblasts cultured on PCL/Fe_3_O_4_ were observed to be synergistically enhanced with an SMF field. Once implanted into calvarial critical‐sized defect in a mouse model, newly formed bone volume was significantly improved by individually the magnetic scaffolds or SMF, and when both magnetic scaffold and SMF combined the bone volume was 2.7 times greater than the control (Figure 14B,C).^[^ ^225^ ^]^ The authors proposed that the osteoblast stimulation could be mediated through integrins‐ and BMP‐mediated signaling pathways. Electrospun scaffolds of polylactic acid (PLA‐HA‐MNP were implanted into rabbit bone defects, and the rabbits then placed into a static magnetic field. Enhanced osteogenesis and faster bone formation were observed when the rabbits were exposed to magnetic field.^[^ ^228^ ^]^ This approach was completely “wireless,” with the magnetic field induced from outside the animal enclosures.

### 5.2 Magnetically Induced Drug Release

**Figure 15:**
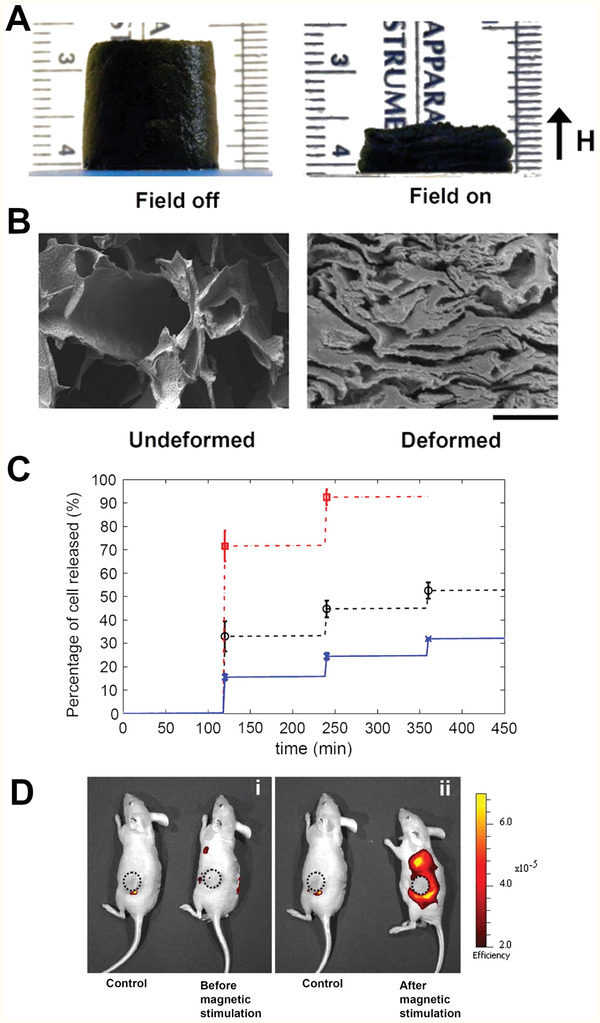
A) A cylinder of a macroporous ferrogel reduced its height ≈70% when subjected to a vertical magnetic‐field gradient of ≈38 A m^−2^. B) SEM images of a freeze‐dried macroporous ferrogel in the undeformed and deformed states. Scale bar: 500 µm. C) Cumulative release profiles of fibroblasts from macroporous ferrogels with 100% (cross), 50% (circle), and 10% (square) of the baseline RGD density, following application of cycled magnetic field. D) In vivo fluorescence images of mice implanted with macroporous ferrogels containing mouse mesenchymal stem cells stained with DiOC18 i) before and ii) after magnetic stimulation. The control case was subject to no magnetic stimulation. The positions of the gel disks are indicated by circles on the figure. Adapted with permission.^[231]^ Copyright 2011, PNAS.

MNP are well known in biomedicine, typically for controlled drug release and delivery with high spatial accuracy as they can be guided and triggered by external magnetic fields. When incorporated into a scaffold MNPs can be used to control drug release, inducing drug release through the structure of the scaffold (i.e., pores opening and closing) in the presence of a magnetic field, or through localized heating within a thermally responsive polymer network to release the molecules.^[^ ^229^ ^]^ A PVA/Fe_3_O_4_ ferrogel was used to encapsulate the model drug B_12_, wherein the scaffold is “closed” when magnetically stimulated due to orientation and aggregation of the MNPs. Once the magnetic field is switched “off,” the pores of the scaffold open and allow the drug to diffuse out.^[^ ^230^ ^]^ As the loaded drug is physically trapped within the material once the magnetic field is applied, this type of scaffold design can also be “refilled” with the target drug for later drug release once again. A ferromagnetic macroporous scaffold demonstrated significant volume change when stimulated by a SMF, creating a compressive deformation of the hydrogel which exerts a water flow out through the interconnected pores of the materials (**Figure** **15**A,B). This facilitates the controlled step‐wise release of molecules across a range of sizes; from the drug mitoxantrone (*M*_r_ 444 444) and plasmid DNA (*M*_r_ 10^6^) down to the chemokine SDF‐1*α* (*M*_r_ 8000). This scaffold was also used to deliver live human fibroblasts in vitro and then mouse MSC in vivo, enabling a controlled release of the encapsulated cells (Figure 15C,D).^[^ ^231^ ^]^ The macrostructure of this material enabled much larger molecules and even cells to be physically released from the hydrogel, and once the magnetic field is removed, the hydrogel can reversibly uptake surrounding liquid and regain the original shape. The use of magnetic field stimulation to control drug release, particularly in a physical approach, is a promising area of controlled release as it can easily be translated in vivo and to implanted biomedical devices. Control can be had not only over the timing of drug release, but also dose through tuning the appropriate magnetic field magnitude for physically pushing the drug out of the scaffold. The use of an implantable macroporous magnetically responsive reservoir of cells is an interesting approach to delivering cells in vivo; controlled release of a cell payload once implanted can be more advantageous compared to waiting for an encapsulating hydrogel to degrade or for the cells to migrate out.

## 6 Conclusion and Future Perspectives

In this review, we have highlighted the unique capabilities of externally applied stimuli to influence stem cell behavior via manipulation of engineered biomaterials. Light‐based, electrical, ultrasonic, and magnetic stimuli each present their own advantages and conversely, limitations, for use as controlled environmental triggers. With regard to controlling biochemical cues, photoresponsive systems benefit from a versatile range of photoinitiated chemistries which enable spatiotemporally controlled covalent linkage and decoupling of bioactive molecules to/from the scaffold material. In contrast, electrochemical, ultrasound, and magnetic systems have found more frequent application for growth factor release in a pulsed or on‐demand release profile, involving stimulated emission from the incorporating scaffold. Electrically and magnetically responsive biomaterials are less capable of spatially precise drug release than focused light or ultrasound systems, as the stimulating energy is conducted throughout the biomaterial (for electroresponsive) or generated by an external field (magnetoresponsive). Photoresponsive systems have also shown a high utility in tuning scaffold mechanical properties, via modulating chemical crosslinking or photomediated degradation. Scaffold structural and mechanical manipulation has also been demonstrated with other modalities such as ultrasound, via physical changes in the scaffold material.

Some stimuli may also be particularly well‐suited for exerting influence toward a specific lineage. Electrical stimulation of conductive materials is often applied in the osteoinductive or myogenic context, as these tissues receive native electrical cues. Magnetic field‐induced mechanical stimulation via embedded nanoparticles has been leveraged frequently for osteogenic induction, whereas light and ultrasound have found use in varied bioactive molecule presentation. Electrical stimulation via biomaterials can also easily be adapted into in vitro or fundamental research through the design of electrode cell culture devices. A researcher's choice of stimuli‐responsive platform for in vitro applications may thus depend on the specific bioactive cue needed for a particular cell type and lineage, as well as the spatiotemporal profile of investigation desired.

Exciting possibilities also exist for combining multiple stimuli in a synergistic fashion to allow for precise control of multiple environmental features. For example, a material could be designed to allow controlled electrical stimulation of cells on a scaffold with precisely photopatterned growth factor cues in order to explore combinatorial stimuli. Another approach could use one stimulus, such as electric field‐induced galvanotaxis, to improve cellular penetration into a particular region of the scaffold, and a second applied stimulus (e.g., light or ultrasound) to provide timed bioactive cues. Precise temporal actuation can be leveraged to ensure that these complex materials allow controlled presentation of variables.

As stimuli‐responsive material platforms continue to evolve in capability and therapeutic capacity, in vivo manipulation of stem cells will be an area of great interest. Development toward in vivo therapeutic use may take different forms depending on the stimulating energy, which each have their own strengths and limitations for in vivo use. Acoustic and magnetic platforms benefit from excellent tissue penetration and are thus highly suited for remotely actuating materials postimplantation, to stimulate bioactive agent delivery or other therapeutic action within the body. Photoresponsive systems, while capable of patterning finer resolution biochemical cue profiles at shallow depths, have more limited signal penetration and focusing capability when applied transdermally which limits use at nonsuperficial tissue depths. Nonetheless, photoactuated platforms still have in vivo clinical potential, particularly for use in superficial locations and could also be used to prepattern a material for subsequent in vivo use. Electrically stimulated platforms may find more use as a pretreatment prior to clinical implantation, as they have demonstrated that short, early time‐point stimulation can influence longer term differentiation. For in vivo applications, while electrical fields can be applied relatively easily in an external set‐up, direct electrical stimulation requires much more invasive approaches to provide a connection to the electroresponsive scaffold. Hence electrical stimulation is more applicable in vitro, through prepatterning the surface presentation of ECM proteins, conditioning a stem cell culture, or priming an implant with electrically applied cyclic strain.

Additionally, as the field of tissue engineering pushes toward personalized and precision medicine, stimuli‐responsive biomaterial systems hold exciting potential for guiding development of patient‐derived organoids and tissue‐engineered disease models. Applied energy sources can be used to modify bioactive cue presentation, mechanical actuation or physical matrix topography, influencing the development of stem cells into tissue‐like structures and cell‐type patterns. Spatial and temporal control of the applied stimulus can allow for controlled development of an initially more‐homogenous stem cell population into heterogeneous lineage and functional specification, recapitulating the complex multicellular patterns of the tissue of interest. Such models could be used to investigate tissue development under normal and diseased conditions, as well as the efficacy of potential therapeutic interventions for individual patients and disease states.

Taken altogether, external stimuli‐responsive platforms for stem cell manipulation have strong potential to drive advances in biomedicine, through both in vivo therapeutic strategies and tissue‐engineered platforms to investigate dynamic cell behavior in health and disease states.

## Conflict of Interest

The authors declare no conflict of interest.
